# Coronary No-Reflow after Primary Percutaneous Coronary Intervention—Current Knowledge on Pathophysiology, Diagnosis, Clinical Impact and Therapy

**DOI:** 10.3390/jcm12175592

**Published:** 2023-08-27

**Authors:** Gjin Ndrepepa, Adnan Kastrati

**Affiliations:** 1 Deutsches Herzzentrum München, Technische Universität München, Lazarettstrasse 36, 80636 Munich, Germany; kastrati@dhm.mhn.de; 2 German Center for Cardiovascular Research (DZHK), Partner Site Munich Heart Alliance, 80336 Munich, Germany

**Keywords:** coronary no-reflow, microcirculation steal syndrome, microvascular obstruction, pathophysiology, therapy

## Abstract

Coronary no-reflow (CNR) is a frequent phenomenon that develops in patients with ST-segment elevation myocardial infarction (STEMI) following reperfusion therapy. CNR is highly dynamic, develops gradually (over hours) and persists for days to weeks after reperfusion. Microvascular obstruction (MVO) developing as a consequence of myocardial ischemia, distal embolization and reperfusion-related injury is the main pathophysiological mechanism of CNR. The frequency of CNR or MVO after primary PCI differs widely depending on the sensitivity of the tools used for diagnosis and timing of examination. Coronary angiography is readily available and most convenient to diagnose CNR but it is highly conservative and underestimates the true frequency of CNR. Cardiac magnetic resonance (CMR) imaging is the most sensitive method to diagnose MVO and CNR that provides information on the presence, localization and extent of MVO. CMR imaging detects intramyocardial hemorrhage and accurately estimates the infarct size. MVO and CNR markedly negate the benefits of reperfusion therapy and contribute to poor clinical outcomes including adverse remodeling of left ventricle, worsening or new congestive heart failure and reduced survival. Despite extensive research and the use of therapies that target almost all known pathophysiological mechanisms of CNR, no therapy has been found that prevents or reverses CNR and provides consistent clinical benefit in patients with STEMI undergoing reperfusion. Currently, the prevention or alleviation of MVO and CNR remain unmet goals in the therapy of STEMI that continue to be under intense research.

## 1. Historical Perspective

Historical records of no-reflow are difficult to track because vascular events developing in early experimental ischemia/reperfusion models, later known as no-reflow, were described well before the no-reflow phenomenon was recognized. The term “no-reflow phenomenon” was coined in 1967 by Guido Majno et al. [1] in a Letter to the Editor in Lancet (September 9th issue, 1967) to describe the inability to reperfuse rabbit brain regions made ischemic by artery ligation despite restoration of blood flow. The work supportive of the proposal of the term “no-reflow phenomenon” was published approximately 6 months later in the February issue of the American Journal of Pathology [2]. Using electron microscopy, the authors described many morphological (cellular) characteristics of the no-reflow at the capillary level, such as capillary obstruction by cellular swelling, bleb formations originating from the endothelial cells, platelet and red cell aggregates and extravascular compression of the microcirculation [2,3]. These histological findings are considered hallmark characteristics of the no-reflow to this day. However, failure to restore tissue reperfusion following restoration of blood flow was described in a number of ischemia/reperfusion animal models before the term “no-reflow phenomenon” was coined. In 1948, Harman [4] provided one of the best descriptions of the no-reflow in skeletal muscle in the right hind legs of albino male rabbits made ischemic by the application of tourniquets. Angiographic and dye studies assessing the rate of penetration and elimination of bromphenol blue from the ischemic muscle showed that blood circulation through the ischemic muscle after the release of occlusion was extremely sluggish. Of note, the study by Harman established a relationship between duration of ischemia and the speed of elimination of dye from the ischemic muscle, provided histological analysis of ischemic lesions such as tightly packed erythrocytes within the capillaries and interstitial fluid accumulation after release of occlusion and excluded an eventual role of thrombi in the genesis of the syndrome. In the subsequent years, the no-reflow phenomenon was described in a number of experimental ischemia/reperfusion models in kidney [5,6], adrenal gland [7], brain [2,3], myocardium [8], and skin [9]. Demonstration of the no-reflow phenomenon in various animals and organs led Majno et al. [1] to suggest that no-reflow after an ischemic insult may be a general phenomenon. In 1974, Kloner et al. [10], while working in the laboratory of Robert Jennings, performed a study specifically designed to characterize coronary no-reflow (CNR) after coronary occlusion in anesthetized dogs subjected to 40 to 90 min ischemia (by clamping the circumflex coronary artery) followed by clamp release and reperfusion. Using electron microscopy, the authors offered the best description of ultrastructural alterations in the vasculature and working myocardium that stand at the pathophysiological basis of CNR to this day [10,11]. The study by Kloner et al. [10] was highly influential and is often considered an inaugural study in the field of CNR.

The early records of no-reflow in humans remain elusive or subject to interpretation. It is highly plausible that no-reflow could have played a role in the genesis of ischemic muscular contractures (called Volkman’s ischemic contracture) after surgical embolectomy of arterial thrombi. In 1934, Jefferson [12] described a case of removal of a clot from the brachial artery two and a half hours after embolic artery occlusion. The clot was successfully removed but the patient rapidly developed a contracture of moderate severity in the forearm flexor muscles. Similar cases of contractures following clot removal from the acutely (embolic) occluded femoral artery as well as demonstration of this phenomenon in experiments with rabbits were reported by Griffiths [13] in 1940. Thrombolytic studies opened the prospect of assessing the CNR in clinical settings. However, interest in CNR in the thrombolytic era was low. In 1985, Schofer et al. [14] were the first to demonstrate CNR by thallium-201 or technetium-99m scintigraphy in patients with acute myocardial infarction (AMI) of the anterior wall after intracoronary thrombolysis. In the restudied patients, the scintigraphic zone of CNR persisted for 2 to 4 weeks after intracoronary thrombolysis. In the subsequent years, CNR was described by angiography in case reports [15,16,17] or in studies with a limited number of patients [18]. In 1989, Wilson et al. [18] described a syndrome characterized by angina, ST-segment elevation and a striking reduction of blood flow in the dilated artery immediately after balloon angioplasty in five patients with acute thrombotic coronary artery occlusions with no visible distal emboli or side branch occlusions. The syndrome lasted for 48 to 80 min and was not reversed by nitroglycerin or thrombolytic drugs. The condition was explained by microvascular constriction caused by release of potent vasoconstrictors from the clot. In 1992, Ito et al. [19] demonstrated CNR in 39 patients undergoing thrombolysis (10 patients) or coronary angioplasty (29 patients) using myocardial contrast echocardiography (MCE). Of note, the study by Ito et al. [19] showed that no-reflow was a predictor of poor functional recovery of the postischemic myocardium. A study of 1919 percutaneous coronary interventions (PCI) performed in early 1990s showed an overall frequency of CNR (defined as Thrombolysis in Myocardial Infarction [TIMI] blood flow < 3) of 2%. However, the frequency of CNR was 11.5% in patients undergoing PCI for AMI and 4% in patients undergoing treatment of saphenous vein grafts [20]. Morishima et al. [21,22] demonstrated an association between angiographic CNR and in-hospital and long-term outcomes including cardiac death in patients with the first AMI treated with PCI. These studies suggested that the increased risk for long-term complications in patients with CNR may be related to adverse left ventricular remodeling associated with CNR. The purpose of this review is to provide an overview of frequency, pathophysiology, diagnostic tools, predisposing factors, clinical impact and principles of therapy of CNR in patients with ST-segment elevation myocardial infarction (STEMI) treated with primary PCI.

## 2. Pathophysiology of CNR

**Figure 1:**
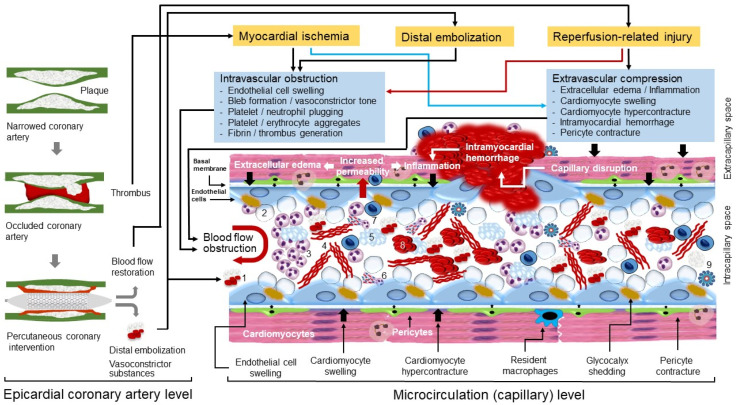
Pathophysiological mechanisms of microvascular obstruction and coronary no-reflow. Pathophysiological events that develop at epicardial coronary artery and microcirculation (capillary) levels are shown. Black arrows denote forces generated outside the capillaries that compress microcirculation, the red arrow points out to increased permeability. Numbers show the following components of microvascular obstruction: 1, atherothrombotic debris; 2, blebs; 3, neutrophil aggregates; 4, fibrin; 5, platelet aggregates; 6, neutrophil extracellular traps (NETs); 7, monocytes; 8; erythrocyte aggregates; 9, inflammasome.

Microvascular obstruction (MVO) is the underlying pathophysiological mechanism of CNR. MVO and CNR after reperfusion of an occluded coronary artery are explained by a joint action of at least four factors: myocardial ischemia, spontaneous or iatrogenic distal embolization, reperfusion-related injury and individual susceptibility (predisposing conditions that increase the odds of developing MVO and CNR). Pathophysiological mechanisms of MVO and CNR are shown in Figure 1.

### 2.1. A Short Description of Myocardial Microcirculation

Coronary microcirculation refers to blood circulation in vessels <200 µm in diameter that are not visualized on coronary angiography. It consists of arterioles, capillaries and venules. All 3 structures participate in the MVO and CNR following ischemia and reperfusion, albeit with different roles. Coronary arterioles have a relatively thick smooth muscle wall, act as resistance vessels and are responsible for keeping a constant precapillary pressure of ~45 mmHg in the setting of autoregulation. In the setting of ischemia/reperfusion injury arterioles contribute to MVO and CNR through impaired vasomotor tone (impaired endothelium dependent vasodilation) and propensity to in situ thrombus formation. Morphometric analyses have shown that there are approximately 2200 capillaries per square millimeter in adult human hearts [23]. It has been estimated that there are approximately 8 million capillaries in the human heart. Coronary capillaries contain ~1/3 of myocardial blood (~45 mL) which moves with an average speed of 1 mm/sec (at resting state) under a hydrostatic pressure of ~30 mmHg [24,25]. In the setting of ischemia/reperfusion injury, coronary capillaries undergo constriction, obstruction, and compression, which lead to a marked reduction in the number of open vessels and diminished delivery of oxygen and nutrients to the surrounding tissue. Coronary capillaries are the main site of clogged microcirculation and MVO occurring as a response to ischemia and/or reperfusion. Coronary venules have a weak smooth muscle and, consequently, manifest weak muscular vascular responses (venular hydrostatic pressure is ~15 mmHg). However, coronary venules participate in the ischemia and/or reperfusion-related MVO by serving as a preferential site of leucocyte and platelet adhesion via expression of adhesion molecules and subsequent inflammation [25,26].

### 2.2. Myocardial Ischemia

Total cessation or drastic reduction (>80%) of coronary blood flow results in severe myocardial ischemia in subtended myocardium. Previous studies that have investigated cardioprotective measures in ischemia/reperfusion models were potentially conceptually flawed in that they focused on the cardiomyocytes paying little attention to microcirculation. This could be one reason of the failure of cardioprotective measures to translate into clinical benefit [27]. Microcirculation—the key component of MVO and CNR—is vulnerable to ischemia and reperfusion injury. In the following material, we focused on the endothelial cells and other components of microcirculation, whereas the impact of ischemia on cardiomyocytes was not addressed.

Endothelial cells are more resistant to ischemia than surrounding cardiomyocytes and may survive hypoxia for minutes to days following the installation of ischemia [10,28]. Endothelial cells are abundant in myocardium representing 3% to 5% of the myocardial volume or approximately 45% of total cells or 60% of nonmyocyte cells in the murine myocardium [29]. Experimental studies using human umbilical vein endothelial cells showed that 75% of cells survived 24 h of hypoxia [30] and >50% of cells survived 48 h of hypoxia [28]. Concentration of high-energy phosphates (adenosine triphosphate [ATP] and creatine phosphate) is low in myocardium and can support contraction for only a few effective systoles. The oxygen present in capillaries (as oxyhemoglobin) and cardiomyocytes (as oxymyoglobin) is exhausted after 8–10 s, which brings to almost total cessation of oxidative phosphorylation and effective myocardial contraction. At 15 to 20 s of ischemia (artery occlusion), anaerobic glycolysis supervenes as the only source of generation of ATP. At 60 s of ischemia anaerobic glycolysis slows markedly and at 40 to 60 min of total ischemia, anaerobic glycolysis essentially stops [31]. The lack of coronary blood flow and cessation of aerobic metabolism lead to accumulation of various catabolites in the cells and interstitial space of ischemic tissue including lactate, protons (tissue acidosis), ammonium, degraded nucleotide phosphates (adenosine diphosphate, adenosine), products of glycogen breakdown (glucose-1-phosphate), glucose-6-phosphate and many other products of intermediary metabolism. Higher concentrations of these catabolites increase the osmotic load within the cells and interstitial space, which generates an osmotic gradient forcing the water to move inside the cells or from intracapillary to interstitial space leading to cellular swelling and interstitial edema [32]. Thus, endothelial cell swelling and interstitial edema are important contributors to MVO and CNR during myocardial ischemia.

Lack of high-energy phosphates leads to severe perturbations in ionic hemostasis in endothelial cells. Apart from further contributing to endothelial cell swelling and intracapillary space obstruction as a response to ischemia, altered ionic hemostasis has other negative actions promoting endothelial cell dysfunction. Ischemic endothelial cells show increased concentration of calcium in cytoplasm, which activates endothelial cell contractile elements [33]. Actin filaments constitute 5–15% of the total protein in endothelial cells [34] and actin cytoskeleton is critical for maintenance of endothelial barrier function [35]. Calcium-induced contraction of endothelial cell filaments reduces mechanical support for endothelial cell membrane promoting cytoplasmic budding or blebbing into the intracapillary space [36]. Bleb formation appears to be further favored by loss of antegrade pulsatile flow and increased shear stress [37]. Blebs and their role in the intracapillary obstruction have been described since inaugural structural studies of no-reflow [2,3]. Calcium-induced filament contraction appear to change the cellular shape, which destabilizes cellular junctions and increase intercellular permeability. There are other factors that destabilize endothelial intercellular junctions during myocardial ischemia. Ischemia-induced expression of vascular endothelial growth factor (VEGF)—a major regulator of vascular permeability [38]—and dissociation of VEGF receptor 2-vascular endothelial (VE)-cadherin (a cell adhesion protein of adherens junctions) complex lead to increased inter-endothelial cell permeability [39]. VEGF activates Scr (a member of Src kinase family) via phosphorylation, which leads to phosphorylation of tyrosine residues of VE-cadherin of the interendothelial cell junctions. This action promotes VE-cadherin internalization and reduces the amount of VE-cadherin in the interendothelial cell junctions [40,41]. The removal of VE-cadherin from the intercellular junctions further destabilizes intercellular connection and increase intercellular permeability. In experimental conditions VE-cadherin phosphorylation is also facilitated by increased shear stress [40]. VEGF also activates endothelial nitric oxide synthase (eNOS) in the caveolae of endothelial cells [42], further contributing to increased vascular permeability. Activated endothelial and circulating cells (platelets and neutrophils) show increased expression of adhesion molecules [43], which may be further potentiated by subsequent reperfusion by thrombolysis or angioplasty [44]. Exposed adhesion molecules mediate platelet and leukocyte endothelial interactions, which facilitate trapping of these cells in the ischemic microvascular space (discussed later under this subheading).

Glycocalyx is perhaps the earliest microcirculation component that is damaged in the course of ischemia/reperfusion. Glycocalyx is an important component of endothelial barrier. Glycocalyx represents a 0.5 µm thick carbohydrate-rich matrix that covers the endothelium surface throughout the capillary system [45]. The thickness of glycocalyx exceeds the length of extracellular domains of most endothelial adhesion molecules [46], which in normal conditions prevents the adhesion of circulating cells to endothelial cells. The highly hydrophilic nature of glycocalyx enables creation of a relatively fixed (albeit exchangeable) water layer on the surface of endothelial cells, which together with electrostatic interactions with circulating erythrocytes, reduces the capillary hematocrit compared with that found in the systemic circulation and facilitates the passage of blood through the capillaries [47]. Glycocalyx is degraded upon exposure to ischemia [48,49], reactive oxygen species (ROS) [49,50], oxidized lipoproteins [51], acute hyperglycemia [52], tumor necrosis factor alpha (TNFα) [53], matrix metalloproteinase (MMP) 2 and 9 [54], inflammatory states and vigorous volume loading [55]. Nitric oxide (NO) appears to be protective against glycocalyx shedding [56]. Glycocalyx degradation (shedding) contributes to MVO and CNR by damaging the capillary barrier and increasing capillary permeability, which contributes to endothelial cell and interstitial edema [57] and by enabling leukocyte [58] and platelet [59] adhesion to endothelial cells facilitating the entrapment of these cells in the intracapillary space.

Platelets and neutrophils are recruited in the capillaries following myocardial ischemia and contribute to ischemic injury, MVO and CNR [60]. Following activation by ischemia, platelets expose their adhesion molecules and aggregate to endothelial cells (facilitated by glycocalyx shedding), neutrophils, erythrocytes and to each other contributing to microcirculation obstruction (Figure 1). These cellular aggregates have been demonstrated in capillaries from the very first ultramicroscopic characterization of no-reflow [2,3]. A significant increase in the neutrophil–platelet aggregates and monocyte–platelet aggregates was shown in the coronary sinus blood samples of microsphere-induced CNR in Yorkshire pigs [61]. Apart from mechanical blockage by aggregates and microthrombi, activated platelets release various biological active substances including nucleotides, proteases, platelet-activating factor (PAF), ROS, adhesive proteins (fibronectin, von Willebrand factor, thrombospondin, P-selectin, glycoprotein IIb/IIIa, fibrinogen), vasoconstrictors such as thromboxane A2 and serotonin, growth factors, coagulation and complement system factors, various cytokines and chemokines, pro-angiogenic factors and microvesicles and exosomes [60,62,63,64,65,66]. These substances contribute to proteolytic destruction of endothelial cells and intercellular junctions (and increased permeability), intracapillary blood coagulation, chemiotaxis and recruitment of leukocytes in microcirculation and promote inflammation, apoptosis and angiogenesis. However, it appears that the degree and nature of platelet contribution to ischemia and reperfusion-related injury depend on the state of platelet activation. Experimental studies in rats and guinea pigs showed that ischemia/reperfusion damage was ameliorated and endothelial integrity was improved by platelet or platelet-derived perfusion [67,68]. Platelet glycoprotein IIb/IIIa receptor blockage has reduced microvascular thrombosis in murine models of acute stroke [69] and platelet depletion counteracts deleterious effects of acute hypercholesterolemia on infarct size and CNR in an ischemia/reperfusion model in rabbits [70]. Glycoprotein IIb/IIIa receptor blockade with abciximab improved the recovery of microvascular perfusion and enhanced the recovery of contractile function in the area at risk in patients with AMI after coronary stenting [71]. Neutrophils are recruited early in the ischemic myocardium [60]. Neutrophils transmigrate through endothelial cells by interaction with endothelial cell junction proteins due to the highly chemotactic milieu in the ischemic microcirculation [72]. Activated neutrophils aggregate with other cells and form neutrophil extracellular traps (NETs) clogging the microcirculation and impeding blood flow [73,74]. Neutrophils are a major source of ROS [75], myeloperoxidase [76] and proteolytic enzymes (such as, elastase and metalloproteinase-9) [77,78], which in turn, promote degradation of all components of capillary barrier (glycocalyx, endothelial cells and basal membrane), leading to vascular leakage, increased vascular permeability and excess edema. Following initial infiltration and activation, neutrophils and other inflammatory cells (monocyte/macrophages and lymphocytes) participate in the powerful local and systemic inflammatory response that develops in patients with AMI.

Growing evidence suggests that pericytes play an important role in the genesis of CNR. With a density of approximately 3.6 × 10^7^ pericytes/cm^3^, pericyte is the second most frequent nonmyogenic cell found in the heart in vitro [79]. Pericytes contract in circumferential and longitudinal directions influencing the diameter and stiffness of capillaries. The close proximity of pericytes to sympathetic axons suggests that their tone may be under noradrenergic regulation [80]. Pericytes have an established role in autoregulation of cerebral blood flow and contribute to vasoconstriction of cerebral capillaries and entrapment of erythrocytes and leukocytes in no-reflow zones following cerebral ischemia [81]. In mouse models of cerebral ischemia, pericytes caused capillary constriction and obstructed erythrocyte passage despite reopening the middle cerebral artery [82]. Cardiac pericytes constrict coronary capillaries and reduce microvascular blood flow after ischemia, despite reopening of the culprit artery [80]. In rat models of ischemia and reperfusion, areas of capillary blockage colocalized with pericytes, which showed a 37% diameter reduction. Notably, intravenous adenosine—a pericyte relaxant drug—increased the capillary diameter by 21% (at pericyte somata), decreased the capillary block by 25% and increased the perfusion volume by 57% [80]. Recent evidence also strongly suggests that pericytes contract as a response to myocardial ischemia and they play an important role in CNR [83,84]. Ischemic preconditioning inhibited the contraction of microvascular pericytes induced by cardiac ischemia/reperfusion injury suggesting that protective role of ischemic preconditioning may be at least partially mediated by its impact on pericyte function [85]. It has been proposed that cardiac pericytes may represent a novel therapeutic target aiming at protection of coronary microcirculation and alleviation of MVO and CNR after AMI [65].

Myocardial ischemia sets into operation a large number of systemic and local vasoconstrictor stimuli that impair the coronary vasodilator reserve and increase the vasoconstrictor tone of microcirculation, which may be alleviated by vasodilator drug therapy [86]. Experimental studies have shown that arterioles undergoing ischemia/reperfusion fail to dilate under the effect of endothelium dependent vasoactive substances acetylcholine and bradykinin, suggesting that endothelium-dependent relaxation of coronary microvessels was markedly impaired during ischemia/reperfusion cycle [87,88]. Although the underlying mechanisms of persistently increased vasoconstrictor tone in microcirculation undergoing ischemia/reperfusion are unknown, excess alpha-adrenergic tone [89,90], angiotensin II [91,92], excessive production of ROS and cytokines-like tumor necrosis factor alpha (TNFα) [93,94], vasoconstrictor substances released from the culprit lesions including serotonin and thromboxane A2 [94], endothelin [95] or neuropeptide Y [96], most likely in combination, do play a role. Reduced availability of NO caused by inhibition [97] or uncoupling of endothelial nitric oxide synthase [98], upregulation of arginase [99,100] and increased production of ROS [101] affect vascular tone (among other deleterious effects) during ischemia/reperfusion. Although increased vasoconstrictor tone was considered deleterious in that it contributes to obstructed microcirculation and MVO, we hypothesize that increased vasoconstrictor tone in the ischemic area may have a protective role as well. By obstructing the microcirculation, the increased vasoconstrictor tone confines ischemic products, necrotic debris and a large number of harmful catabolites and active substances to the ischemic area, preventing them from spreading to surrounding viable myocardium or from entering the circulation. This may be at least one reason why vasodilator therapy fails to improve clinical outcome, although it apparently may improve reperfusion. In addition, vasodilator therapy may preferentially dilate vessels surrounding ischemic region and shift the blood towards viable surrounding myocardium (microcirculation steal syndrome) worsening the reperfusion of ischemic region, apparently associated with improved reperfusion, at least by angiographic (TIMI flow grade) markers. However, these hypotheses need testing.

In aggregate, myocardial ischemia leads to various alterations affecting all components of microcirculation leading to various degrees of MVO and CNR. Endothelial cells, however, are relatively resistant to ischemia and may remain ultrastructurally intact up to 6 h of ischemia in anesthetized cats [102]. In rat models of AMI, no clear damage to capillary endothelium occurred after 30 min of ischemia without reperfusion and no reduction of inter-endothelial cell junctions was observed after 90 min occlusion of left anterior descending artery [103]. Another study in mongrel dogs subjected to ligation of the circumflex branch showed mild swelling of endothelial cells but no totally occluded capillaries following prolonged periods of ischemia. The study suggested that ischemia-induced loss of vascular competence was unlikely to be due to intravascular thrombosis, endothelial cell swelling, or external compression by interstitial edema [104]. Thus, shorter periods of ischemia (≤1 h) are characterized by mild edema with almost no (or little) signs of cellular necrosis, inflammation or capillary injury. Longer periods of ischemia (≥2 h) are characterized by a uniform infarcted area (cellular death), marked infiltration by inflammatory cells (primarily neutrophils), and severely damaged capillaries and evident hemorrhage [54].

### 2.3. Distal Embolization

Distal embolization of atherothrombotic fragments from the atherosclerotic plaque occurs spontaneously or during the primary PCI procedure as a result of guide-wire passage, lesion preparation and stent implantation. Angiographically visible distal embolization is documented in 11% to 17% of primary PCI procedures in patients with STEMI [105,106,107,108]. However, the true incidence of lesser degrees of distal embolization appears to be much higher, with one study showing visible debris in 73% of patients who received a distal embolization protection system [109]. Histologically, the embolized material consists of a mixture of atheromatous debris, platelet aggregates, erythrocytes, fibrin, cholesterol crystals and inflammatory cells [110,111,112,113]. Distal embolization is more frequent in atherosclerotic plaques with large volumes (particularly plaques with large necrotic core) [114] and those with more thrombus at the lesion site [115,116]. Moreover, erythrocyte-rich thrombi, elevated glucose level on admission, larger culprit vessel, pre-balloon dilation and right coronary artery as culprit lesion have been identified as independently associated with a higher risk of distal embolization during the primary PCI procedures [108,111]. Although most studies have assessed distal embolization in patients with STEMI characterized by a large thrombus burden, it has been suggested that distal microembolization may occur during plaque erosion at the culprit lesion in patients presenting with non-STEMI [110]. Since microthrombi preferentially end in well reperfused and viable myocardium (directed by blood stream), distal embolization kills potentially salvageable myocardium [117]. One experimental study in dogs suggested that embolizing particles tend to flow away from the central infarcted area (forced by developing CNR) and accumulate in the infarct border contributing to infarct extension [118]. The study suggested that embolizing particles are more important for infarct expansion than for CNR at least in the early phase of reperfusion. Distal embolization contributes to CNR, increases biomarkers of myocardial necrosis, causes patchy microinfarcts that disproportionally impair the left ventricular function beyond the actual amount of damaged myocardium, increases infarct size and is associated with a poor clinical outcome [41,107,118,119]. Although CNR may develop in the absence of distal embolization, distal embolization may promote (or aggravate) MVO and CNR by a number of mechanisms (recently reviewed by Kleinbongard and Heusch [110]) including mechanical obstruction, increased vasoconstrictor tone at arteriolar and microcirculation levels by soluble vasoconstrictor substances contained in embolized material [94,95,96], generation of a prothrombotic milieu and favorization of platelet aggregation and in situ thrombosis [110,120] and inducing a powerful local inflammatory response [110,121]. Experimental studies of microthrombi-induced CNR in Yorkshire pigs showed that distal embolization was associated with elevated levels of metalloproteinase-2 and a reduction in the activity of survival kinase (Akt) within the infarct zone 3 days after AMI, with both events helping to explain deleterious effects of distal embolization on infarct size [122]. Atheromatous debris fraction of the embolized material is extremely resistant to antithrombotic or thrombolytic agents used to treat patients with AMI and the only way to clear it is through inflammation or other processes set into operation to clear necrotic tissue after AMI.

### 2.4. Reperfusion-Related Injury

Endothelial cells and microcirculation are extremely sensitive to reperfusion-related injury—a condition first described by Jennings et al. [123] in 1960 in canine hearts. There are at least five manifestations of reperfusion-related injury: reperfusion-induced arrhythmias, myocardial stunning, MVO, intra-myocardial hemorrhage and reperfusion-induced cell death (or lethal reperfusion injury) [124,125]. Although, myocardial ischemia and reperfusion appear to be opposite events in terms of blood interruption and restoration, they are similar in terms of molecular and cellular events that develop following both events. It appears that reperfusion-related injury completes the cellular damage initiated by ischemia. However, the motif of reperfusion-related injury is unclear but it may represent an early scavenger mechanism to clear ischemia-induced irreversibly damaged cells.

Experimental studies in dogs by Kloner et al. [10] showed that fluorescent dye thioflavin S managed to penetrate ischemic myocardium after 40 min of ischemia followed by reperfusion. However, after 90 min of ischemia followed by reperfusion, thiofalvin S failed to penetrate the ischemic myocardium and perfusion defects were observed in subendocardium. Reperfusion failure was observed within seconds of clamp release and it was well established within the first few minutes. Notably, perfusion defects were always found within the ischemic-necrotic zone but not in the surrounding viable myocardium not undergoing ischemia/reperfusion, strongly suggesting that CNR is due to microvascular damage within the zone of necrosis [10]. Later studies showed that if proximal coronary arteries were occluded for a longer time (3 h), then perfusion defects were more widespread and reached mid-myocardium and occasionally the outer layers of myocardium [126]. These studies showed that the extent of CNR depends on the duration of ischemia, a finding that has been confirmed in the clinical studies as well [127]. The most consistent histologic finding was demonstration of areas of swollen endothelium and formation of intraluminal membrane-bound protrusions or blebs that obstructed the capillary lumen. Other (less frequently observed) markers of microcirculation damage included loss of pinocytotic vesicles, endothelial gaps, rupture of capillary walls with extravasation of red blood cells, deposits of fibrin tactoids in vicinity of endothelium gaps, platelet–leukocyte aggregates, and rouleaux structures of erythrocytes. The local edema involving endothelium and surrounding myocardium suggested an initial restoration of some blood flow, which was later interrupted by reperfusion-induced MVO and swollen cardiomyocytes. Occasionally a capillary compressed (and consequently obstructed) by two swollen cardiomyocytes was seen. Reperfusion-induced hypercontracture of myocardium was also involved in the compression of microcirculation [128,129]. Studies by Ambrosio et al. [130] in open-chest dogs subjected to 90 min occlusion of left circumflex coronary artery followed by reperfusion for 2 min or 3.5 h showed that the extent of the CNR area grows over the reperfusion time. Thus, the area of impaired reperfusion (absent thioflavin) was 9.5% of the initial area at risk in animals reperfused for 2 min and 25.9% of the area at risk in dogs reperfused for 3.5 h. Importantly, serial measurements using microspheres showed that areas with adequate reperfusion at 30 min of reperfusion had a marked fall of perfusion at 3.5 h of reperfusion. Another study in dogs undergoing 90 min (balloon) occlusion of the left anterior descending coronary artery followed by reflow showed that the extent of MVO (assessed by hypo-enhanced regions on contrast-enhanced cardiac magnetic resonance [CMR]) increased threefold over the 48 h after reperfusion (3.2%, 6.7% and 9.9% of the left ventricular mass at 2, 6, and 48 h, respectively) [131]. Similar findings were reported by Reffelmann and Kloner [132] in a rabbit model of reperfusion. The area of CNR increased progressively from 12.2% after 2 min of reperfusion to 30.8% after 2 h of reperfusion and to 34.9% of the initial area at risk after 8 h of reperfusion. The expansion of CNR zone was fastest within the first 2 h of reperfusion, finally encompassing ~80% of the infarct size. Regional myocardial blood flow was hyperemic at 2 min of reperfusion, decreased later and remained unchanged (plateau) between 2 and 8 h of reperfusion. Notably, no hemorrhage was visible after 2 min of reperfusion but it reached a value of 37.3% of area at risk after 8 h of reperfusion. One study that included patients with the first AMI showed that MCE-defined CNR present at 24 h after reperfusion was sustained at 1 month in approximately 50% of the patients [133]. These studies strongly suggested that CNR is primarily a reperfusion injury-related phenomenon.

One of the earliest events that develop following blood flow restoration to ischemic myocardium is exacerbation of ischemia-initiated interstitial and cellular edema. An experimental study in dogs undergoing a 90 min balloon occlusion of left circumflex artery followed by 60 min reperfusion showed increased wall thickness in the reperfused myocardium due to tissue edema eventually leading to CNR because of mechanical compression [134]. CMR imaging studies in pigs [135] and humans [136,137] have shown a bimodal pattern of myocardial edema following reperfusion. The early wave of edema appears to be due to exposure of a hyperosmotic interstitium (due to accumulation of catabolites produced during ischemia) to normo-osmotic blood at reperfusion. The early wave of edema occurs immediately after reperfusion and markedly diminished at 24 h as a result of catabolite washout from the interstitium [135]. The second (late) wave of edema develops gradually following ischemia/reperfusion and is maximal around day 7 following reperfusion [135]. The second wave of edema is explained by increased vascular permeability related to influx of inflammatory cells and healing process of the infarcted tissue [135].

Ischemia-initiated endothelial cell edema is further accentuated by reperfusion. Upon restoration of blood flow, the extracellular pH is rapidly restored, which stimulates the Na^+^/H^+^ exchanger and Na^+^/HCO3^-^ symporter leading to proton extrusion from the cells, rapid normalization of intracellular pH, massive Na^+^ influx, and intracellular Ca^2+^ overload [36,124]. The increased Ca^2+^ in endothelial cells leads to cellular retraction and intercellular gap formation and blebbing resulting in increased vascular permeability and obstruction of intracapillary space. In addition to cellular swelling, increased ATP availability upon restoration of blood flow, restored intracellular pH and abundant cytoplasmic Ca^2+^ favor the hypercontracture of cardiomyocytes and contraction band formation—a histological marker of reperfusion [138] further compressing the microvasculature. One of the most important deleterious effects of reperfusion is mitochondrial injury related to opening of mitochondrial permeability transition pore (MPTP)—a nonselective channel that enables movement of water, ions and low molecular weight solutes across the inner mitochondrial membrane. The MPTP channel remained closed during ischemia under the inhibitory effect of acidosis and increased Ca^2+^ content [125,139]. The MPTP opening leads to loss of mitochondrial inner membrane potential, oxidative phosphorylation/ATP production uncoupling, mitochondrial membrane rupture, release of apoptotic factors (cytochrome c) and cell death by necrosis [36,124].

Blood cells contribute to reperfusion-related microvasculature injury and MVO. Serial CMR imaging studies in pigs have shown that interstitial edema is maximal immediately after reperfusion, whereas the maximal content of neutrophils, macrophages, and collagen is observed at 24 h, 4 days and 7 days after reperfusion [135]. Neutrophils brought to ischemic microcirculation upon blood restoration are activated and tend to aggregate with other neutrophils, platelets or endothelial cells leading to microcirculation plugging and MVO. Activated neutrophils produce inflammatory cytokines, ROS, elastase and metalloproteinases, which cause capillary destruction, vascular leakage and a strong inflammatory response. Newly brought platelets are activated in the highly prothrombotic microcirculation undergoing ischemia/reperfusion, and activated platelets aggregate causing further capillary plugging and release numerous active substances with vasopressor and prothrombotic effects further increasing vascular tone and microthrombi formation (see: Myocardial ischemia). Thus, newly arrived cells in previously ischemic microcirculation are activated and recruited in capillaries and further accentuate ischemia-induced MVO and CNR.

Intramyocardial hemorrhage is one of the most severe manifestations of reperfusion-related injury that is closely linked with MVO and CNR [140,141]. Experimental studies in anesthetized dogs [142] and autopsy studies in patients with AMI (recanalized with intracoronary streptokinase within 3.5 h of ischemia) [143] showed that intramyocardial hemorrhage is always observed following reperfusion but not in nonreperfused infarctions. Intramyocardial hemorrhage is always confined to the necrotic zone, predominantly in the central part of the necrosis, and tends to diminish towards the border zone [143,144]. Serial imaging studies in pigs have shown that the hemorrhage score (from 0 absent to 5 very severe) was 0 at 120 min, 2 at 24 h, 4 at day 4 and 1 at day 7 after the reperfusion [135]. CMR imaging and histological studies in dogs undergoing 4 h of coronary occlusion followed by 1 h of reperfusion showed that CMR-assessed hemorrhage size (decreased signal intensity zones) correlated closely with hemorrhage size defined by histology (correlation coefficient of 0.96) [145]. In dogs without reperfusion, no macroscopic or CMR-defined zones of hemorrhage were observed [145]. Marked increase in permeability and gap formation (of sufficient diameter to allow passage of erythrocytes) in the vascular barrier after ischemia followed by reperfusion may lead to extravasation of red blood cells in the perivascular space. Platelet-activating factor (PAF)—a potent inflammatory mediator [146]—and prolonged adhesion of neutrophils to endothelium may promote gap formation [147] via basal membrane destruction and endothelial cell detachment via released active proteases [147] and formation of neutrophil extracellular traps (NETs) [74,148]. Intramyocardial hemorrhage is more common after prolonged severe ischemia followed by reperfusion, which results in necrosis of endothelial cells, breakdown of basal membrane and destroyed microvessels [149,150]. These studies support the notion that intramyocardial hemorrhage represents the most severe form of ischemia/reperfusion. Microvascular destruction and local consumption of coagulation factors due to coagulation cascade activation and intravascular microthrombi formation promoted by activated endothelium and inflammation have also been suggested as mechanisms for extravasation of erythrocytes and hemorrhage in areas of MVO after AMI [151]. Apart from being a manifestation of severity of ischemia/reperfusion after AMI, intramyocardial hemorrhage per se aggravates MVO and CNR through extracellular compression and other mechanisms. CMR imaging studies in swine undergoing circumflex coronary artery occlusion for 75 min by a balloon catheter and in patients with AMI showed an overlap and a close anatomic correlation between areas of intramyocardial hemorrhage and MVO (correlation coefficients of 0.85 and 0.87, respectively) [151]. In addition, CMR imaging studies showed that all patients with AMI and intramyocardial hemorrhage on T2* imaging had CMR-confirmed MVO [152] or that 80% of patients with MVO had CMR evidence of intramyocardial hemorrhage [153]. Intramyocardial hemorrhage is irreversible and induces a powerful and prolonged inflammatory response. Extravasated erythrocytes undergo destruction, which leads to iron release and deposition in the infarct zone. CMR imaging studies in canine models of ischemia/reperfusion showed that intramyocardial hemorrhage leads to iron deposition in the infarct zone up to 2 months after the acute event and that newly recruited macrophages colocalize with iron deposits, suggesting a prolonged inflammatory burden in the chronic phase of myocardial infarction [154]. Intramyocardial hemorrhage appears to be more frequent after reperfusion by thrombolytic agents than primary angioplasty. In a series of 19 necropsies of patients undergoing thrombolytic therapy, 74% of infarcts treated with thrombolytic agents (but none of the infarcts undergoing balloon angioplasty alone) were hemorrhagic [155]. Intramyocardial hemorrhage appears to be associated (or be more frequent) with larger infarct size, greater MVO, larger left ventricular dimensions, lower left ventricular ejection fraction (LVEF), anterior wall infarct location and glycoprotein IIb/IIIa inhibitor use. However, after adjustment, only anterior wall infarct location and the use of glycoprotein IIb/IIIa inhibitors were associated with higher odds of intramycardial hemorrhage [156]. Intramyocardial hemorrhage is a determinant of infarct size, infarct expansion and reduced myocardial salvage after reperfusion [157]. Intramyocardial hemorrhage is a strong correlate of adverse outcomes, which is stronger than infarct size [152] and patients with MVO and intramyocardial hemorrhage have a worse prognosis than patients with MVO without intramyocardial hemorrhage [158].

Ischemia/reperfusion is associated with a strong inflammatory response in the infarct zone, predominantly mediated by neutrophils, which contributes to MVO and CNR. Monocytes, macrophages and lymphocytes do participate in the inflammatory response, as well. One experimental study in mongrel dogs, in which a segment of a large epicardial coronary artery was deprived of blood flow for 3 h followed by reperfusion, showed an influx of neutrophils within the media of ischemic/reperfused vessels but not in the nonischemic vessels. Electron microscopic analysis showed that neutrophils were often located between the endothelial cells and the elastic lamina in the ischemic/reperfused vessels [159]. The deleterious effects of neutrophils (and other immune cells) are predominantly mediated by release various active substances including inflammatory cytokines [160], MMPs (particularly MMP-9) [77,78], ROS [75,98] and myeloperoxidase [76]. The necrotic debris (among other stimuli) activates the NLRP3 inflammasome, which regulates caspase-1 activity, stimulates production (and release) of large amounts of cytokines (primarily IL-1β and IL-18) and promotes inflammatory cell death via pyroptosis [161]. Inflammasome contributes to MVO and CNR by exacerbating endothelial cell damage and vascular leakage (promoting interstitial edema and microcirculation compression), heightening vasopressor tone and promoting cellular trapping and stasis and microthrombi formation in microcirculation [162,163]. Patients with AMI who develop MVO and CNR have higher levels of several inflammatory cytokines in circulation, including C-reactive protein [127,164], interleukin 6 (IL-6) [165] and interleukin 8 (IL-8) [166] compared with patients who did not develop these phenomena. Patients developing CNR have significantly higher levels of myeloperoxidase at culprit lesions than patients without CNR [167,168]. Myeloperoxidase produces a large number of highly reactive species which attack all known cellular components leading to reduced NO availability, endothelial dysfunction and impaired vasoreactivity [76]. The role of inflammation in tissue healing is outside the scope of this review.

Of all mechanisms and mediators proposed to date to explain microcirculation damage in the setting of ischemia/reperfusion, ROS and MMPs have received most attention for their role in the genesis of MVO. Excess production of ROS and activation (or overexpression) of MMPs are underlying mechanisms of tissue damage (including microcirculation) during ischemia/reperfusion and their actions appear to be mutually dependent. In the setting of ischemia/reperfusion, ROS appear to have multiple cellular sources (endothelial cells, platelets, neutrophils or other immune cells and resident macrophages) and the main producers at the molecular level are xanthine oxidase, NADPH oxidase, mitochondrial electron transport chain and uncoupled NO synthase [54,98]. Excess amounts of ROS interact with any biological structure in their vicinity, rendering them dysfunctional. ROS are involved and play a critical role in almost all cellular and molecular events leading to MVO in the setting of ischemia/reperfusion. Activated MMPs appear to have multiple sources including endothelial cells, smooth muscle cells, inflammatory cells and resident macrophages [54]. MMPs have a wide specificity and cleave a wide range of extracellular matrix components including glycocalyx, inter-endothelial cell junctions, basal membrane and extracellular substrates such as adhesion molecules, cytokines and chemokines [54]. MMPs play a critical role in the MVO by participation in the increased vascular permeability and leakage, glycocalyx shedding, capillary destruction and intramyocardial hemorrhage. Detailed information on the biology of ROS and MMPs and their role in genesis of MVO during ischemia/reperfusion are provided in 2 excellent reviews by Granger and Kvietys [54,98].

## 3. Diagnosis and Frequency of CNR

CNR is highly dynamic in nature, develops gradually (over hours) following coronary blood flow restoration and persists over days to weeks depending on severity, duration and extent of myocardial ischemia and application of therapeutic measures aiming to prevent or alleviate ischemia/reperfusion injury. The diagnostic yield of any method used to diagnose CNR depends on the extent and severity of CNR and the timing of examination. Transient slowing of restored myocardial blood flow or small under-reperfused myocardial segments may go undetected whereas fixed MVO developing over an extensive myocardial area/volume is more reliably detectable. The use of diagnostic methods early (before CNR has developed) or late (after CNR has resolved) after the restoration of epicardial blood flow may fail to detect (or underestimate) CNR. These factors as well as the sensitivity of the method per se used to diagnose CNR may explain the wide variations in the frequency of CNR across the studies. Before analyzing specific methods used to diagnose CNR in clinical setting, two concepts may need clarification. First, the term CNR is used to describe coronary blood flow stasis or MVO after all PCI procedures including elective PCI in patients with chronic coronary syndromes. However, since CNR is an ischemia/reperfusion syndrome resulting from the sequence of coronary artery occlusion (acute ischemia) and reopening (blood flow restoration-related reperfusion injury), blood slowing detected immediately after elective PCI in patients without an acute antecedent (or ongoing) myocardial ischemia may not be CNR. In this scenario, no-flow may be caused by clogged microvasculature by distal embolization of atherosclerotic/thrombotic material in the course of PCI or pre-existing MVO. Second, the definition of CNR requires restoration of blood flow without flow-impeding obstacles at the large coronary artery level. Thus, massive and angiographically visible distal embolization of atherosclerotic/thrombotic material occurring during the primary PCI procedures simply shifts the mechanical obstacle to blood flow from the epicardial artery to a more distal location and, as such it may not represent a true CNR phenomenon.

CNR after primary PCI may be diagnosed using invasive (coronary angiography and catheter-based coronary physiology measurements) and noninvasive (MCE, electrocardiogram and CMR imaging) methods. Diagnostic methods used to detect CNR in the clinical setting differ widely with respect to their sensitivity to detect the condition and the most optimal timing of their use to diagnose CNR remains unknown.

Coronary angiography is routinely used to diagnose CNR following PCI. There are at least four angiographic metrics that have been used to diagnose CNR: Thrombolysis in Myocardial Infarction (TIMI) flow grade, corrected TIMI frame count, myocardial blush grade and TIMI myocardial perfusion grade. TIMI flow grade estimates blood flow in the epicardial coronary arteries. Blood flow is quantified using a scale between 0 and 3 and a TIMI flow grade of <3 is used to diagnose CNR. TIMI flow grade has prognostic value and failure to restore a TIMI flow grade of three was independently associated with increased risk of mortality after PCI in patients with acute coronary syndromes (ACS) [169]. CNR has been diagnosed in up to 32% of patients with STEMI by TIMI grading criteria [170]. Although, the assessment of CNR using TIMI flow grade is convenient and simple, it does not reflect tissue reperfusion (or microvascular function) and thus, the method lacks sensitivity to diagnose CNR [171,172]. A study from our group showed that tissue reperfusion assessed by myocardial perfusion grade was not fully restored (myocardial perfusion grade ≤ 2) in 34% of patients with a TIMI flow grade of three after primary PCI [173]. Thus, the definition of CNR as a TIMI flow grade of <3 is highly conservative and leads to underestimation of CNR in a high proportion of patients undergoing primary PCI. Corrected TIMI flow count represents the number of frames required for the dye to reach a standardized distal landmark after correction for the vessel (epicardial artery) length [174]. Faster (lower) corrected TIMI frame count was associated with improved in-hospital and one-month outcomes after thrombolysis [175] and better functional recovery after successful primary angioplasty in patients with AMI [176]. Corrected TIMI frame count correlated inversely with peak blood flow velocity by intracoronary Doppler but not with the degree of microvascular injury after primary angioplasty in patients with the first STEMI of anterior wall [177]. These data suggest that corrected TIMI frame count reflects epicardial blood flow but not microcirculation.

Myocardial blush grade or the degree of myocardial contrast staining following intracoronary contrast injection is also used to assess myocardial microvasculature and tissue reperfusion after primary PCI [178]. Myocardial blush grade is scaled between 0 (no contrast staining) to 3 (contrast staining similar to that of contralateral non-infarcted myocardium) and a myocardial blush grade of <3 indicates CNR. However, myocardial blush grade represents a low contrast-to-noise ratio imaging and depends on the operator’s experience, which may lead to unacceptably high interobserver variability and suffers from the same limitations as the TIMI flow grade method [41,179]. These characteristics limit the usefulness of myocardial blush grade to assess CNR after primary PCI. Post-procedural TIMI flow grade or myocardial blush grade do not necessarily correlate with the presence of MVO detected by CMR imaging in patients with STEMI undergoing successful primary PCI [180,181]. Although myocardial perfusion grade appears to correlate with one-year mortality even in patients with TIMI flow grade of 3 [182], it was discordant with ST-segment resolution in up to 40% of patients after primary PCI [183]. One study has shown that MVO assessed by CMR but not TIMI flow grade or myocardial blush grade correlated with the left ventricular function following primary PCI [184].

TIMI myocardial perfusion grade is also used to assess myocardial reperfusion based on the densitometry of contrast entry, duration, and clearance from the ischemic myocardium [179]. It is scaled between 0 (no tissue reperfusion) and 3 (minimally persistent myocardial contrast staining following three cardiac cycles of washout) and a TIMI myocardial perfusion grade of ≤2 indicates impaired tissue reperfusion and CNR. TIMI myocardial perfusion grade appears to be superior to other angiographic indices with respect to the assessment of tissue reperfusion in reperfused patients with STEMI. A combination of TIMI flow grade with TIMI myocardial perfusion grade (a TIMI flow grade of ≤2 or a TIMI flow grade of 3 with a TIMI myocardial perfusion grade of 0–1) diagnosed CNR in 29% of patients with STEMI treated successfully with primary PCI [185]. Impaired TIMI myocardial perfusion grade correlated with MVO assessed by CMR imaging at 3 to 4 days [186,187], infarct size at 7 days and 3 months [188] and LVEF at 90 days [187] after STEMI. Impaired TIMI myocardial perfusion grade (≤2) correlated with biochemical markers of myocardial necrosis and increased risk of death, myocardial infarction or ischemic events on Holter monitoring by 48 h in patients with non-ST-segment elevation ACS undergoing PCI [189]. In aggregate, although achieving favorable coronary angiography indices are associated with an improvement of prognosis after primary PCI and should be strived for, with the exception of TIMI myocardial perfusion grade, these indices are poor correlates of MVO and CNR assessed by sensitive CMR imaging. One reason for the low sensitivity of angiographic methods to detect CNR may be related to the timing of coronary angiography, which may be well ahead of development of CNR. Since CNR is a dynamic process that develops hours to days following blood flow restoration to previously ischemic myocardium, angiographic indices obtained at the end of primary PCI procedure reflect the impact of ischemia and distal embolization on microcirculation but not the most important factor of CNR, i.e., reperfusion-related injury. 

MCE uses gas-filled microbubbles, which are very effective in scattering of ultrasound and an ideal tracer of microcirculation. Microbubbles have rheological properties similar to the red blood cells and their size (<5 μm) allows them to pass through capillaries without blocking them. Microbubbles remain entirely within the vascular space and myocardial contrast intensity following intravenous or intracoronary microbubble injection reflects their concentration in the microvascular compartment of myocardium [190,191]. The microbubble lingering inside the myocardium or the lack of contrast opacification in the echocardiograms obtained following intravenous or intracoronary microbubble injection indicate CNR and MVO. MCE can localize the zone of MVO and quantify its extent within the infarcted myocardium [192]. The prevalence of CNR diagnosed by MCE varies between 37% [193] and 66% [133]. MCE is a validated method to assess myocardial reperfusion [194,195] and the CNR diagnosed by this technique correlates closely with adverse left ventricular remodeling and poor prognosis after AMI [19]. MCE is limited by moderate spatial resolution and dependence on operator’s experience [65], poor echocardiographic window preventing reliable measurements in 8% of patients [196] and an inability to detect vasodilatation or vasoconstriction within a previously ischemic myocardial area [192].

CMR imaging is the most sensitive technique to detect MVO and diagnose CNR in the clinical setting. By providing multislice views, with a high spatial resolution, CMR imaging enables an accurate quantification and localization of MVO within the infarcted area as well as transmural extent of CNR and necrosis (infarct size) within the infarcted region. CMR imaging also detects the hemorrhagic transformation and extravasation of erythrocytes within the infarct core, which represent a frequent component of CNR that portends a poor prognosis [191]. Contrast-enhanced CMR is based on the differences in the distribution of the contrast agent (gadolinium chelate injected intravenously) depending on the status (degree of injury or obstruction) of microcirculation within the injured vs. healthy myocardium [41]. CMR imaging-based detection of MVO (and consequently CNR) is defined as the lack of contrast uptake during the first pass of contrast agent (imaging obtained within one minute after injection), the lack of early gadolinium enhancement (imaging obtained within 2–3 min after injection) or the lack of late gadolinium enhancement (imaging 10–15 min after contrast injection) [197]. On the first pass imaging, MVO typically appears as a central dark zone within an area of early enhanced myocardium indicating a focal absence of contrast enhancement within the infarcted area [198]. Since gadolinium may diffuse slowly over time from the CNR zone, the area of MVO may become smaller in late gadolinium enhancement imaging, which may explain the diagnosis of MVO in a higher proportion of patients on the first pass imaging compared with late gadolinium enhancement imaging [198]. However, it has been suggested that MVO on late gadolinium enhancement imaging is a better correlate (prognostic marker) of subsequent left ventricular remodeling and adverse cardiovascular events than MVO seen on the first pass gadolinium imaging [199,200]. Using CMR imaging, MVO was diagnosed in up to 95% of patients with STEMI and restored TIMI flow grade of 3 during the first pass contrast-enhanced imaging [201] and 57% of patients with STEMI within 7 days after primary PCI by late gadolinium enhancement imaging [202]. Using CMR imaging, MVO was diagnosed in 13.8% of patients with non-ST-segment elevation myocardial infarction [203] as well as in patients with myocardial infarction with nonobstructive coronary arteries (MINOCA) [204,205]. CMR imaging typically is performed hours to days after reperfusion of patients with STEMI. Consequently, early events developing after epicardial blood restoration and the relationship between CNR and infarct size (or myocardial salvage) cannot be assessed by this technique.

Several other diagnostic methods have been used to assess CNR in clinical setting. The degree and speed of ST-segment elevation resolution in the post-reperfusion electrocardiogram correlates closely with tissue reperfusion and MVO after primary PCI. A rapid and complete (>70%) ST-segment elevation resolution after primary PCI indicates prompt (and complete) restoration of tissue reperfusion [206], which is associated with markedly reduced frequency of MVO or CNR on MCE [207] or CMR imaging [181]. In a study by Nijveldt et al. [181], residual ST-segment elevation was the only independent predictor of microvascular injury after adjustment in multivariable analysis. ST-segment resolution in the intracoronary electrocardiogram recorded at the time of procedure correlated with CMR-assessed MVO 4 days after reperfusion in 64 patients with STEMI undergoing primary PCI [187]. Although standard electrocardiogram is cheap and readily available, it lacks sensitivity to diagnose CNR and there is no consensus on the most appropriate electrocardiographic leads or timing of electrocardiogram recording after reperfusion. MVO and CNR can be assessed using invasive (catheter-based) coronary physiology indices. Several physiological indices that characterize coronary microcirculation may be obtained such as coronary flow velocity patterns, coronary flow reserve, index of microvascular resistance, hyperemic microvascular resistance, resistive reserve ratio, instantaneous hyperemic diastolic flow velocity-pressure slope and coronary zero flow pressure [62]. In brief, microvascular injury (and consequently MVO) is characterized by shortening of diastolic deceleration time and presence of systolic retrograde flow, decrease in coronary flow reserve, an increase in hyperemic microvascular resistance index, an increase in the index of microcirculatory resistance, a decrease in the coronary conductance by the instantaneous hyperemic diastolic velocity pressure slope index and an increase in zero-flow pressure [62,191] (for details see Konijnenberg et al. [62] and Lanzer et al. [191]). Although these indices offer a good characterization of microvascular injury (and MVO) after reperfusion and may be applied in the catheterization laboratory, they mostly remain as research tools and are not routinely used to diagnose MVO and CNR in patients with STEMI. Although myocardial scintigraphy offered the first evidence on the existence of CNR in humans [14], nuclear imaging techniques—single photon emission tomography (SPECT) and positron emission tomography (PET) with various perfusion tracers—have limited use for assessing CNR in current practice due to problems related to technical difficulties, costs, patient’s radiation, and availability of other highly sensitive imaging techniques to detect CNR, particularly CMR imaging. Representative diagnostic studies of CNR are shown in Table 1.

In aggregate, CNR following reperfusion of patients with AMI should be searched for and diagnosed. Coronary angiography-based techniques are most convenient, but they are performed too early after reperfusion (before full-scale CNR has developed) and, consequently, they underestimate CNR. CMR imaging is the most sensitive technique to diagnose CNR that provides information on its extent and location within the infarcted myocardium in clinical setting. Apart from CNR diagnosis and characterization, CMR imaging also detects intramyocardial hemorrhage and accurately estimates infarct size, further improving the risk stratification of patients with AMI. Other techniques (electrocardiogram, MCE, and nuclear imaging) have limited use for the diagnosis of CNR in current clinical practice.

## 4. Individual Susceptibility to CNR

The frequency of CNR after primary PCI differs widely. Aside from sensitivity of the method used to detect CNR and the timing of the assessment (already discussed), a number of other factors appear to predispose to CNR after primary PCI including, infarct size, pre-existing endothelial and microvascular dysfunction, cardiovascular risk factors, culprit lesion characteristics, invasiveness of coronary intervention and genetic predisposition to CNR.

Several experimental [130,208] and clinical [127,209] studies have shown that infarct size is a determinant of CNR. A higher frequency of CNR has been reported in clinical conditions that lead to larger infarct size, such as culprit lesion location in the proximal left anterior descending artery [209,210] and longer time-to-treatment interval [127,211]. One study by our group identified advanced age, no smoking, previous myocardial infarction, Killip class, serum creatinine, C-reactive protein, time-to-treatment interval, LVEF, baseline TIMI flow and scintigraphic initial perfusion defect as correlates of CNR. After adjustment, only four variables, previous myocardial infarction, baseline TIMI flow, C-reactive protein and initial perfusion defect, correlated independently with a higher risk of CNR after primary PCI [127]. The incidence of CNR is markedly higher in patients with STEMI compared with patients with non-ST-segment elevation or patients undergoing elective PCI [212].

Pre-existing endothelial and microvascular dysfunction are common in patients with AMI and they likely contribute to MVO and CNR after primary PCI [213]. Coronary artery segments distal to the atherosclerotic plaques undergo remodeling with hypertrophy of vascular wall and attenuation of vasomotor responses [214]. Diseases associated with (or predisposing to) coronary microvascular dysfunction and the underlying mechanisms of this association have been reviewed [215]. Indeed, pre-existing coronary endothelial and microvascular dysfunction increase the susceptibility of microcirculation to ischemia/reperfusion-related injury and facilitate the development of MVO and CNR [216,217].

Culprit lesion morphology appears to impact on frequency of CNR after primary PCI. Atherothrombotic plaques responsible for ACS are larger and softer (contain more lipids, inflammation and thrombus) and are more prone to be fragmented (and embolized) during coronary interventions [117]. One study showed that a large lipid index (defined by optical coherence tomography) and plaque burden (defined by intravascular ultrasound) were associated with a higher risk of CNR after primary PCI [218]. Furthermore, a long target lesion length, larger reference diameter, and high thrombus burden on angiography or large vessels with lipid pool-like image on ultrasound imaging are reported to be independent correlates of CNR [219,220]. One angiographic study of patients with AMI identified the cutoff pattern of occlusion (an abrupt cutoff without taper before the occlusion) in the infarct-related artery, accumulated thrombus (>5 mm) proximal to the occlusion, presence of floating thrombus, persistent contrast stasis distal to the obstruction, reference lumen diameter of infarct related artery ≥ 4 mm, and incomplete obstruction with presence of accumulated thrombus more than three times the reference lumen diameter of infarct-related artery as independent correlates of slow flow or CNR [221]. It has been reported that atherectomy and coronary stenting more frequently cause plaque fragmentation and embolization compared with balloon angioplasty [222]. The SYNTAX score can identify patients at risk for CNR with patients with a SYNTAX score of >21 having a 2-fold higher risk for CNR than those with SYNTAX score ≤ 21 [223]. The intervention in saphenous grafts, high pressure balloon inflation and debulking devises appear to increase the frequency of CNR, potentially due to more frequent distal embolization [224]. A large study based on the National Cardiovascular Data Registry (NCDR) identified longer lesion length, more class C lesions, bifurcation lesions and impaired preprocedural TIMI flow as independent angiographic correlates of CNR [225]. Various biomarkers including blood cell-related markers (leukocyte and neutrophil count, mean platelet volume), thromboxane A2, markers of myocardial necrosis (creatine kinase and cardiac troponin), markers of inflammation (C-reactive protein and fibrinogen), Von Willebrand factor, tissue factor, natriuretic peptides and endothelin have been reported to be associated with CNR after primary PCI [226]. The incidence of CNR after primary PCI appears to be higher in patients with elevated uric acid level [227], impaired renal function [228,229], higher systemic immune-inflammation index [230], higher PRECISE-DAPT score [231], lower vitamin D levels [232], higher red blood cell distribution width [233] and higher soluble suppression of tumourigenicity 2 [234].

Although diabetes mellitus is associated with generalized endothelial [235] and microvascular [236,237] dysfunction, and it greatly contributes to poor outcomes in patients with cardiovascular disease [238], patients with STEMI with pre-existing diabetes mellitus did not have more frequent (or extensive) MVO compared with patients without diabetes [239,240]. However, CMR-imaging studies have shown that acute hyperglycemia is associated with MVO in patients with STEMI [239,241,242]. One prospective study that included patients with first STEMI without diabetes showed that hyperglycemia on admission was an independent correlate of presence and size of MVO [243]. It has been suggested that hyperglycemia predisposes to MVO and CNR via a number of mechanisms including leukocyte plugging in capillaries, platelet activation (increased procoagulability), elevated catecholamine level (common in large infarcts), elevated free fatty acid levels and toxic metabolites impairing endothelial function, impaired endothelial-dependent vasodilatation, increased oxidative stress and inflammatory cytokines [241,244]. Although arterial hypertension is commonly associated with endothelial dysfunction (impaired endothelium-dependent vasodilatation), mostly related to reduced availability of NO [245], it was not associated with invasive intracoronary parameters used to assess reperfusion injury in patients with AMI [246] or CMR-defined MVO [246,247]. However, arterial hypertension showed a questionable association (*p* = 0.059) with intramyocardial hemorrhage [246]. The association between hypercholesterolemia and CNR remains poorly investigated and controversial. In patients with normal coronary arteries and arterial hypertension, hypercholesterolemia was associated with depression of both basal and pharmacologically stimulated bioavailability of NO [248] suggesting a role of these conditions in promoting endothelial dysfunction. An experimental study in rabbits fed by 2% cholesterol-enriched diets for 3 days showed that hypercholesterolemia was associated with larger infarct size and larger nonreperfused (no-reflow) zones suggesting that hypercholesterolemia increased infarct size by vascular obstruction [249]. However, one MCE-study found no difference in the incidence of CNR according to hypercholesterolemia in 293 patients with AMI undergoing successful primary PCI [250]. Another CMR imaging study showed modest association (adjusted odds ratio of 1.02) between elevated low-density lipoprotein (LDL) cholesterol level and microvascular injury in 235 patients with STEMI undergoing primary PCI [251]. A recent lipidomics study showed that phosphatidylcholine, alkylphosphatidylcholine, and sphingomyelin, were significantly elevated in plasma of patients with STEMI who developed CNR after primary PCI [252]. Despite well-known deleterious effects of smoking on vasculature, including endothelial dysfunction, vascular remodeling, increased thrombogenicity and proinflammatory action [253], recent CMR imaging studies in reperfused patients with STEMI did not show a significant difference in the presence or extent of MVO in smokers vs. nonsmokers [254,255,256]. However, smoking appears to favor intramyocardial hemorrhage [254,256]. A higher CHA2DS2-VASc Score is also associated with the increased risk of CNR after primary PCI [257,258]. Pre-infarction angina appears to reduce the incidence of CNR, potentially due to salutary effects of ischemic preconditioning [259,260].

There appears to be a genetic predisposition to CNR. A 2007 case-control study showed that survivors of AMI who developed CNR after PCI had more compact fibrin network and resistance to lysis [261], suggesting a genetic predisposition to CNR mediated by genetic factors that modulate fibrin clot properties. Patients with STEMI who developed CNR after primary PCI had higher serum levels of SCUBE1 [signal peptide-CUB (complement C1r/C1 s)-EGF (epidermal growth factor)-like domain-containing protein 1]—a protein expressed in platelets and endothelial cells that could function as an adhesion molecule [262]. The 1976 T/C polymorphism of the adenosine 2A receptor gene may increase susceptibility to microvascular injury and CNR [213]. Single nucleotide polymorphisms in the VEGFA—vascular endothelial growth factor A—and CDKN2B-AS1 genes are associated with abnormal coronary flow reserve and microvascular dysfunction in patients without significant obstructive coronary artery disease referred for cardiac catheterization [263]. Furthermore, there appear to be sex-specific differences with genetic variation in alleles of MYH15 (Myosin Heavy Chain 15), VEGFA, and NT5E (5′-Nucleotidase Ecto) genes increasing the risk of coronary microvascular dysfunction in men [263]. Patients with MVO after primary PCI show a sustained increase in the levels of platelet gp91phox (NOX2)—the catalytic subunit of NADPH oxidase—and 8-iso-PGF2α—a marker of lipid peroxidation—suggesting platelet-mediated ROS generation involvement in MVO [264]. However, the role of genetic factors in pathophysiology of MVO and CNR remains to be better defined.

## 5. Impact of CNR on Clinical Outcome

CNR can be clinically silent or manifest as angina, ST-segment (re)elevation, sudden hemodynamic deterioration while in the catheterization laboratory, malignant ventricular arrhythmias, early congestive heart failure (or cardiogenic shock) and cardiac death [265]. At longer term, patients with AMI developing CNR after the reperfusion are prone to develop depressed left ventricular function, adverse left ventricular remodeling, incident or worsening of congestive heart failure and have reduced survival. In patients with the first AMI of anterior wall, Ito et al. [193] showed that MCE-defined CNR was associated with progressive increase in the left ventricular end-diastolic volume and early and more prolonged congestive heart failure in the postinfarction period with 3 of 47 patients with CNR dying of pump failure, strongly suggesting an association of CNR with subsequent adverse left ventricular remodeling. In 4264 patients with AMI undergoing primary PCI, Resnic et al. [266] showed that CNR was highly predictive of postprocedural MI and in-hospital death. In 599 patients with STEMI undergoing primary PCI, Brosh et al. [267] showed that CNR occurred more frequently after coronary stenting and required balloon pump counterpulsation more often. Patients with CNR had larger enzymatic infarct size, more frequent moderate-to-severe left ventricular dysfunction and higher 6-month mortality than patients without CNR. In a large NCRD data study, patients with CNR showed larger infarct size (creatine kinase-MB: 133 vs. 76 ng/mL) and a higher incidence of in-hospital complications after PCI for AMI including mortality (12.6% vs. 3.8%), reinfarction (2.4% vs. 0.7%), cardiogenic shock (7.4% vs. 1.7%) and heart failure (5.2% vs. 2.1%) compared with patients without CNR [225]. The length of hospital stay was longer in patients who developed CNR compared with patients who did not (mean: 5.9 days vs. 4.9 days) [225]. Of note, CNR was associated with worse outcomes in patients with STEMI and non-STEMI, but the incidence of all complications was higher in patients with STEMI compared with patients with non-STEMI.

Long-term studies have further confirmed a worse long-term prognosis associated with CNR. Morishima et al. [22] assessed the association between CNR and prognosis in 120 patients with the first AMI treated by PCI over a mean follow-up of 5.8 years. CNR was associated with a higher risk of cardiac death, all-cause death, malignant arrhythmias and congestive heart failure. After adjustment, CNR remained an independent correlate of cardiac death and adverse cardiac events. Survivors with CNR had higher end-diastolic and end-systolic left ventricular volumes and plasma brain natriuretic peptide levels and lower LVEF strongly suggesting a role of CNR in adverse left ventricular remodeling. In the study by Bolognese et al. [268] that included 124 patients with AMI, microvascular dysfunction assessed by intracoronary MCE was the only independent correlate of cardiac death and combined adverse events (cardiac death, reinfarction, and heart failure) over a mean 46-month followup. Notably, left ventricular volumes increased progressively and were significantly higher at 6 months in patients with microvascular dysfunction compared with patients without microvascular dysfunction. Our group [127] assessed the association of CNR with myocardial salvage (defined by repeat scintigraphy), left ventricular function at 6 months and mortality at 1 year in 1140 patients with STEMI undergoing primary PCI. CNR was associated with significantly larger initial perfusion defect and final infarct size and lower myocardial salvage by primary PCI (salvage index: 34% of the initial perfusion defect in patients with CNR vs. 55% of the initial perfusion defect in patients without CNR). Notably, patients with CNR had a lower LVEF than patients without CNR (47.7 ± 13.1% vs. 54.2 ± 13.9%) and less LVEF improvement at 6 months and higher mortality (16.7% vs. 5.5%) at 1 year. Our group [185] also studied the association between CNR and 5-year mortality in 1406 patients with STEMI undergoing primary PCI. Patients with CNR had significantly higher scintigraphic infarct size (15% of the left ventricle vs. 8% of the left ventricle) and higher 5-year mortality (18.2% vs. 9.5%). After adjustment, CNR remained significantly associated with the risk of 5-year mortality (adjusted hazard ratio of 1.66). Of note, CNR after primary PCI provided prognostic information that was independent of and beyond the information provided by the infarct size.

Transient CNR, defined as CNR during the primary PCI procedure in the absence of flow limiting obstructions at the culprit lesion that reverses to normal blood flow at the end of the procedure, is also associated with clinical outcomes [269,270,271,272]. In the Primary Angioplasty in Myocardial Infarction (PAMI) trial, transient CNR occurred in 16 patients (1.3%) [269]. Patients with transient CNR had higher in-hospital (13% vs. 2%) and 6-month (31% vs. 3%) mortality. One study that compared transient CNR with persistent CNR reported a lower in-hospital mortality in patients with transient CNR [270]. A retrospective study of 414 patients with STEMI undergoing primary PCI showed a graded increase in the 6-month mortality in patients exhibiting slow flow (6.8%), transient CNR (14.1%) and persistent CNR (44.4%) [271]. Finally, in a large registry that included 4329 Korean patients with AMI (2668 patients with STEMI), the incidences of transient and persistent CNR were 5% and 1%, respectively. In patients with restored flow, transient and persistent CNR, the 3-year all-cause and cardiac mortality was 18.1%, 24.4% and 46.7% and 7.9%, 13.1% and 40.0%, respectively [272]. Transient CNR was associated with higher long-term mortality compared with patients with restored flow and with a lower long-term mortality compared with patients with persistent CNR after primary PCI. The differences in mortality were mostly driven by differences in cardiac mortality. Transient CNR appears to correlate with the characteristics of culprit lesion. A study of patients with stable CAD, in whom the coronary plaques were assessed using the multidetector spiral computed tomography, showed that a low density plaque by computed tomography and reduced LVEF were identified as independent correlates of transient CNR [273]. Likewise, in patients with AMI undergoing PCI, Iijima et al. [274] showed that vessel area, plaque burden in the culprit lesion, ruptured plaque, lipid-like images and thrombus formation by intravascular ultrasound were significantly more frequent among patients with transient CNR than patients with reflow. However, after adjustment, only plaque burden and thrombus formation were independently associated with transient CNR. The underlying mechanisms of transient CNR remain unclear. Coronary blood fluctuations during the primary PCI procedure may reflect vasoconstriction caused by release of vasoactive substances like thromboxane A2 and serotonin when thrombus is squeezed against the artery wall during balloon inflation [275]. Furthermore, embolized fresh loose thrombotic material causing temporary vessel occlusion may be dissolved upon flow restoration facilitated by excess amounts of antithrombotic/anticoagulant drugs used during the primary PCI procedures. Finally, acute recruitment of vessels in the periphery of ischemic/necrotic region may restore TIMI flow grade by blood shifting towards myocardium surrounding the ischemic/necrotic region leading to a microcirculation steal syndrome. However, whether transient CNR at the end of primary PCI procedure heralds the development of persistent CNR remains to be explored.

The underlying mechanisms of the association of CNR with poor outcomes are unclear. Infarct size—a well-known correlate of poor outcome after primary PCI for STEMI—is a strong correlate of CNR. In this regard, CNR is a manifestation of severe ischemic damage of the myocardium characterized by prolonged ischemia affecting extensive parts of the myocardium. Factors that predispose to CNR (see: Individual susceptibility to CNR) also predispose to a poor clinical outcome, regardless of CNR. Although infarct size is closely related to CNR, whether CNR per se impacts on the infarct size remains controversial. Since, the zone of CNR is almost invariably located within the dead tissue, it has been suggested that CNR does not cause ischemic cell death [129]. Clinical studies in patients with STEMI have shown that CNR is associated with larger final infarct size independent of initial infarct size and reduced myocardial salvage by primary PCI [127]. By impeding blood flow to necrotic tissue, CNR delays the removal of necrotic debris and impedes the arrival of cells and cytokines that are involved in the tissue healing [129]. Experimental studies in rats showed that CNR persists up to one month after reperfusion and is associated with scar thinning and infarct expansion [276]. Likewise, clinical studies have shown that MVO and CNR are most important correlates of left ventricular adverse remodeling after reperfusion [268,277]. Finally, intramyocardial hemorrhage is common in patients with CNR [153,157]. As already mentioned, intramyocardial hemorrhage is associated with larger infarct size and infarct expansion after reperfusion [157] and is an independent correlate of major adverse cardiovascular events after reperfusion in patients with STEMI [278].

## 6. Therapy of CNR

Despite more than 50 years of research, little progress has been made in finding a treatment strategy of proven efficacy to be routinely used to prevent CNR in patients with STEMI. Many therapies that showed benefits in reducing MVO and CNR in animals failed to result in similar benefits in clinical setting in patients with STEMI. Although some therapies improved some markers of reperfusion, so far, no therapy applied to prevent or alleviate MVO or CNR has resulted in clinical benefit in terms of reduction of hard clinical endpoints such as mortality. All pathophysiological mechanisms of CNR—distal embolization, myocardial ischemia and reperfusion-related injury—and predisposing factors (when possible) have been targeted by nonpharmacological or pharmacological preventive strategies as single or combined strategies. These therapies were applied before, during or after cardiac catheterization and primary PCI procedure to prevent or alleviate CNR (when it develops).

### 6.1. Therapy against Distal Embolization

Nonpharmacological and pharmacological therapies have been used to reduce the incidence of distal embolization in the setting of primary PCI procedures in patients with STEMI. Intuitively, direct stenting (stenting without predilation) was expected to reduce MVO and CNR by reducing distal embolization. Early randomized studies [279,280] with limited numbers of patients and meta-analyses [281,282] suggested that direct stenting may reduce the incidence of CNR in patients with STEMI. In one meta-analysis, however, direct stenting was significantly better in reducing CNR in nonrandomized studies but not in randomized trials [281]. A recent study including patient-level data from three randomized trials showed no benefit of direct stenting in improving the markers of tissue reperfusion including ST-segment resolution or myocardial blush grade and no direct stenting-by-thrombus aspiration interaction with respect to ST-segment resolution or myocardial blush grade [283].

Distal protection devices have been used to reduce distal embolization of atherothrombotic debris during the primary PCI. The EMERALD (Enhanced Myocardial Efficacy and Recovery by Aspiration of Liberated Debris) trial randomized 501 patients with STEMI to PCI plus balloon occlusion and aspiration distal microcirculatory protection system or PCI alone. Distal protection system failed to reduce CNR and had no significant impact on final TIMI flow, final corrected TIMI frame count, myocardial blush grade, ST-segment resolution > 70%, infarct size or 6-month incidence of major adverse cardiovascular events (MACE) [109]. In other randomized trials, routine use of distal protection devices during the primary PCI did not improve microvascular perfusion, limit infarct size or reduce the occurrence of MACE [284,285]. Distal protection devices are no longer recommended to be used as adjunct to primary PCI.

Mechanical or aspiration thrombectomy devices have also been used to reduce distal embolization in the setting of primary PCI. A 2013 meta-analysis showed that aspiration thrombectomy reduced the risk of MACE and all-cause mortality with no impact on final infarct size or LVEF. With respect to markers of tissue reperfusion, aspiration thrombectomy improved ST-segment resolution and TIMI blush grade. Mechanical thrombectomy did not improve TIMI blush grade, infarct size or the incidence of MACE; however, it improved ST-segment resolution at 60 min [286]. The meta-analysis discouraged the use of mechanical thrombectomy but it offered some evidence that aspiration thrombectomy may protect microcirculation and improve clinical outcome when used as adjunct to primary PCI. Two large, randomized trials showed no benefit of aspiration thrombectomy in the setting of primary PCI [287,288]. The Trial of Routine Aspiration Thrombectomy with PCI vs. PCI alone in patients with STEMI (TOTAL) study showed no difference in the incidence of angiographic CNR between aspiration thrombectomy and PCI alone groups (2.4% vs. 2.8%; *p* = 0.28). However, it showed a significant increase in the incidence of stroke with aspiration thrombectomy [288]. These studies showed that aspiration thrombectomy does not protect microcirculation or reduce the incidence of CNR or improve clinical outcome when used as adjunct to primary PCI in patients with STEMI. 

A strategy of deferred stenting—a two-step strategy of initial reperfusion by balloon angioplasty (or thrombus removal) followed by stent implantation hours or days thereafter—was used with the hope that it would reduce the rate of vessel dissection or distal embolization. In a study of 101 patients with STEMI with ≥1 factor for CNR, deferred stenting reduced the frequency of CNR (2% vs. 14%) and improved myocardial salvage assessed by CMR at 6 months [289]. However the DANAMI-3-DEFER trial that included 510 patients with at least one CMR study showed that deferred stenting did not reduce infarct size or the extent or MVO (43% vs. 42%) or increase myocardial salvage compared with conventional primary PCI [290]. A 2018 meta-analysis of randomized trials with 1570 patients showed that a strategy of deferred stenting reduced the incidence of slow flow or CNR (8.8% vs. 16.6%) but not MVO assessed by CMR (48.4% vs. 52.6%; *p* = 0.51) [291]. The treatment effect for slow flow or CNR and MVO correlated with a thrombus score grade > 3 at the baseline angiography and with the total stent length in the culprit vessel.

Mesh-covered stents have been developed to prevent distal embolization by trapping and excluding embolism-prone material at the level of culprit lesion in patients with STEMI. The MASTER (Safety and Efficacy Study of MGuard Stent After a Heart Attack) trial, which randomized 433 patients with STEMI to a mesh-covered stent (MGuard stent) or a bare-metal or drug-eluting stent, showed that the mesh-covered stent improved complete ST-segment resolution ≥ 70% and postprocedural TIMI flow grade of three with no significant impact on infarct size assessed by CMR or 30-day incidence of MACE [292]. Patients implanted with MGuard stent had a significantly higher one-year incidence of MACE driven by ischemia-driven target lesion revascularization [293]. One study has suggested that MGuard stent may be beneficial in patients with a high-thrombus burden [294]. In one observational study, pressure controlled intermittent coronary sinus occlusion (PICSO) lowered the index of microcirculatory resistance in patients with STEMI and higher values of this metric before PCI (>40) and the CMR-assessed infarct size at 6 months [295]. This approach may reduce MVO by redistributing venous blood towards ischemic border, which may enhance the wash-out of catabolites from the ischemic region [215].

Glycoprotein 2b/3a receptor inhibitors were used to reduce thrombotic events including distal embolization and improve microcirculation due to their inhibitory effects on platelet aggregation. However, clinical results with these agents have not been convincing. In the On-TIME-2 (Ongoing Tirofiban in Myocardial Infarction 2) trial, routine prehospital high-bolus dose of tirofiban improved ST-segment resolution before and one hour after PCI [296]. The trial also showed that prehospital tirofiban reduced the incidence of MACE (death, recurrent myocardial infarction, urgent target vessel revascularization or thrombotic bail-out) at 30 days [296]. A study of 162 patients developing angiographic CNR (defined as TIMI flow grade < 3) after primary PCI randomized to receive intracoronary tirofiban (25 µg/kg) or placebo (0.9% isotonic saline solution) showed that intracoronary tirofiban improved TIMI flow grade and restored normal reperfusion in 26% of patients and in-hospital MACE were significantly lower in the tirofiban group (19% vs. 36%; *p* = 0.013) [297]. A recent study that included 226 patients with STEMI assigned to receive intravenous or intracoronary tirofiban showed that intracoronary tirofiban reduced the frequency and extent of MVO (36% vs. 56%; *p* = 0.004), improved left ventricular end-diastolic volume at 6 months but had no effect on the MACE rate at 1 year compared with intravenous tirofiban [298]. In the Intracoronary Abciximab and Aspiration Thrombectomy in Patients With Large Anterior Myocardial Infarction (INFUSE-AMI) trial, intracoronary abciximab but not aspiration thrombectomy, was associated with a significant reduction of infarct size by CMR at 30 days in patients with large anterior STEMI presenting early after symptom onset and undergoing primary PCI with bivalirudin anticoagulation. However, intracoronary abciximab was not associated with better tissue reperfusion as assessed by final TIMI flow grade, myocardial blush grade or complete (>70%) ST-segment resolution after PCI [299]. The AIDA STEMI (Abciximab Intracoronary vs. intravenous Drug Application in STEMI) showed no difference in the 90-day incidence of MACE between patients assigned to intracoronary vs. those assigned to intravenous abciximab; however, the incidence of new congestive heart failure was lower with the intracoronary administration of the drug [300]. The CMR substudy of the AIDA STEMI trial, showed no difference between the two strategies with respect to final infarct size, MVO, intramyocardial hemorrhage or LVEF at one-week CMR imaging [172]. Glycoprotein 2b/3a inhibitors appear to increase the risk of intramyocardial hemorrhage in patients with STEMI, which was associated with a poor outcome [156]. Current guidelines gave a class IIa, level of evidence C, for the use of glycoprotein 2b/3a inhibitors for patients with STEMI and CNR [301].

In summary, therapy against distal embolization has failed to produce beneficial clinical effects in a consistent manner. Although the reasons why this therapy failed to prevent or ameliorate CNR are unclear, two putative mechanisms may be offered: first, therapy (in particular mechanical devices) may fail to prevent distal embolization or even may facilitate it at the time of device placement, and second, distal embolization may play a modest role in the pathophysiology of MVO and CNR.

### 6.2. Pharmacological Therapy

A large number of pharmacological agents or therapeutic strategies, alone or in combination, have been used to reduce infarct size, boost myocardial salvage and prevent or reduce reperfusion injury and MVO or CNR after primary PCI in patients with STEMI. Apart from antithrombotic (aspirin, clopidogrel, prasugrel or ticagrelor) and anticoagulant (unfractionated heparin or bivalirudin) drugs that are routinely used during the primary PCI, numerous drugs including, statins, angiotensin-converting enzyme inhibitors, calcium channel blockers, beta-blockers, antioxidants (recombinant human superoxide dismutase, desferoxamine, edaravone and allopurinol), neutrophil and complement system inhibitors (pexelizumab), antidiabetic drugs (exenatide), glucose–insulin–potassium infusion, antiischemic agents (trimetazidine), inhibitors of mitochondrial permeability transition pore opening (cyclosporine), NO donors, Na^+^/H^+^ ion exchanger inhibitors (cariporide), Na^+^/Ca^2+^ ion exchanger inhibitors (caldaret), K^+^ATP channel agonists (nicorandil), atrial natriuretic peptides, erythropoietin, adenosine, endothelin receptor inhibitors, protein kinase C inhibitors (delcasertib) as well as therapeutic strategies of hypothermia, hyperoxemia and ischemic conditioning have been used to prevent MVO and CNR [302]. The most commonly used drugs to protect microcirculation, prevent MVO and CNR or reduce infarct size are shown below.

Adenosine—a purine nucleoside and a potent vasodilator of arterioles and microcirculation via binding to A2 receptors—has been tested as a means to prevent (or reverse) MVO and CNR after primary PCI. Experimental studies suggested that adenosine ameliorates ischemia/reperfusion injury, limits infarct size and improves left ventricular function. Earlier studies showed that intravenous adenosine reduces infarct size when used as adjunct to thrombolytic drugs or primary PCI although with a neutral effect on clinical outcomes in patients with STEMI [303,304]. The Intracoronary Nitroprusside Versus Adenosine in Acute Myocardial Infarction (REOPEN-AMI) trial showed that intracoronary high dose of adenosine, but not nitroprusside improved MVO assessed by ST-segment resolution. The angiographic MVO (TIMI flow grade ≤ 2 or 3 with a myocardial blush grade < 2) or MACE (a composite of cardiac death, myocardial infarction, target lesion revascularization, and heart failure requiring hospitalization) at 30 days were not improved by adenosine [305]. A randomized trial by Desmet et al. [306] showed that selective high-dose intracoronary adenosine-infused distal to the culprit lesion failed to promote myocardial salvage or reduce MVO in patients with STEMI. Similarly, the REperfusion Facilitated by LOcal adjunctive therapy in STEMI (REFLO-STEMI) trial found no significant difference in CMR-assessed infarct size or MVO in patients allocated to intracoronary high-dose adenosine, sodium nitroprusside or controls. On per-protocol analysis, however, the infarct size and the rate of MACE at 30 days and 6 months were increased and LVEF was reduced in the adenosine arm compared with control arm [307]. A recent meta-analysis of 26 randomized controlled trials with 5843 patients with ACS undergoing revascularization showed that intracoronary or intravenous adenosine offered no clinical benefit in terms of reduction of MACE, all-cause mortality, nonfatal myocardial infarction or heart failure. In patients undergoing PCI, adenosine reduced myocardial blush grade 0–1 and TIMI flow grade 0–2 but had no effect on infarct size or LVEF. Of note, adenosine increased the risk of atrioventricular block and supraventricular and ventricular arrhythmias is studies with ischemia time > 3 h [308]. Evidence from these studies does not support the use of adenosine as adjunctive to primary PCI.

Sodium nitrite—a NO donor—is a powerful arteriolar vasodilator with antiplatelet and inti-inflammatory properties. The Nitrates in Acute Myocardial Infarction (NIAMI) trial randomized 229 patients with STEMI to receive either an intravenous infusion of 70 μmol sodium nitrite or matching placebo. The drug failed to reduce the infarct size measured by CMR at 6–8 days. In addition, there were no significant differences between nitrite and placebo with respect to the area under the curve of troponin I and creatine kinase, left ventricular volumes or ejection fraction measured at 6–8 days and infarct size measured at 6 months [309]. In the REOPEN-AMI trial, a complete (≥70%) ST-segment resolution was observed in 71% of patients assigned to adenosine, 54% of patients assigned to nitroprusside and 51% in patients assigned to saline (*p* = 0.75 for nitroprusside vs. saline) [305]. Based on these data, there is no proven benefit of intravenous nitrite or nitroprusside as a means to prevent or treat MVO and CNR after primary PCI.

Calcium channel blockers (verapamil, diltiazem and nicardipine) have been used to treat CNR, but the quality of studies is poor [310]. A 2015 meta-analysis of eight randomized controlled trials with 494 patients showed that intracoronary verapamil/diltiazem injection significantly reduced the frequency of CNR [311]. In a retrospective study of 72 patients with ACS, intracoronary nicardipine reversed CNR (defined as restoration of TIMI flow grade of 3) in 71 patients (98.6%) with no adverse hemodynamic or chronotropic effects [312]. Intracoronary infusion of a cocktail consisting nicardipine and adenosine, appears to reduce the frequency of CNR during rotational atherectomy [313]. However, the low quality of studies does not allow any solid recommendation for the use of calcium channel blockers for prevention of CNR in patients with STEMI.

The impact of beta-blocking agents on MVO and infarct size in patients with STEMI remains controversial. One study in Yorkshire pigs showed that intravenous metoprolol was associated with a 5-fold higher myocardial salvage and a significant improve in LVEF compared with placebo [314]. This effect was at least partially explained by metoprolol-induced modulation of inflammatory response by inhibiting neutrophil migration and neutrophil–platelet aggregate formation [315]. Preclinical studies have also shown that carvedilol and nebivolol may protect microcirculation and reduce the frequency of CNR and infarct size. One ischemia/reperfusion study in swine showed that carvedilol reduced the area of CNR and infarct size possibly due to reduced plasma and tissue endothelin-1 levels by carvedilol-induced activation of the K_ATP_ channels [316]. Another study in mice with extensive AMI of anterior wall showed that nebivolol improved endothelium-dependent vasorelaxation, (potentially via NO-mediated improvement of endothelial function), inhibited cardiac NADPH oxidase activation after AMI and improved left ventricular dysfunction at 4 weeks [317]. The Effect of Metoprolol in Cardioprotection During an Acute Myocardial Infarction (METOCARD-CNIC) trial showed that early pre-reperfusion intravenous metoprolol reduced infarct size estimated by CMR and (slightly) improved LVEF in 270 patients with STEMI of anterior wall presenting within 6 h from pain onset [318]. However, in the larger Early Beta-blocker Administration before reperfusion primary PCI in patients with ST-elevation Myocardial Infarction (EARLY-BAMI) trial that randomized 683 patients with STEMI presenting within the first 12 h in Killip class I to II without atrioventricular block, intravenous metoprolol did not reduce infarct size estimated by CMR at 30 days or improve LVEF compared with placebo [319]. These data do not support the early intravenous beta-blockage to protect microcirculation, promote myocardial salvage or reduce infarct size in patients with STEMI undergoing primary PCI.

Statins were thought to improve microcirculation and prevent or treat CNR through their pleiotropic effects including endothelium protection, microcirculation dilatation and antithrombotic and anti-inflammatory actions. The Efficacy of High-Dose AtorvaSTATIN Loading Before Primary Percutaneous Coronary Intervention in ST-Elevation Myocardial Infarction (STATIN STEMI) trial showed that high-dose (80 mg) atorvastatin pretreatment improved angiographic MVO (corrected TIMI frame count and myocardial blush grade) and ST-segment resolution but not MACE at 30 days compared with low (10 mg) atorvastatin dose [320]. In the study by Hahn et al. [321] that randomized patients with STEMI undergoing primary PCI to receive atorvastatin 80 mg before PCI and for 5 days after PCI or 10 mg atorvastatin daily after PCI, there was no significant difference between the groups with respect to scintigraphic infarct size, myocardial blush grade 2/3 or complete ST-segment resolution at 60 min after PCI. The Statins Evaluation in Coronary Procedures and Revascularization (SECURE-PCI) trial that randomized 4191 patients with ACS evaluated with coronary angiography to proceed with a PCI to receive two loading doses of 80 mg of atorvastatin or matching before and 24 h after a planned PCI showed that periprocedural use of loading doses of atorvastatin did not reduce the risk of MACE at 30 days. In the subgroup of patients with STEMI, atorvastatin reduced the incidence of MACE but without treatment effect interaction [322]. These studies do not support the periprocedural use of statins as means to protect microcirculation, reduce MVO or promote myocardial salvage in patients with STEMI undergoing primary PCI.

Intracoronary fibrinolysis has been used to prevent or treat MVO and CNR. In the Trial of Low-dose Adjunctive alTeplase During prIMary PCI (T-TIME), intracoronary alteplase at doses of 10 mg and 20 mg did not reduce MVO compared with placebo [323]. However, the trial was stopped prematurely due to futility reasons. A recent meta-analysis of six randomized controlled trials with 890 patients showed that intracoronary fibrinolysis did not improve postprocedural TIMI flow 2 or 3, but it improved complete ST-segment resolution and was associated with a trend for fewer in-hospital MACE events with no difference in bleeding events compared with placebo [324]. The Bivalirudin Infusion for Ventricular InfArction Limitation (BIVAL) study tested whether bivalirudin reduces infarct size compared with unfractionated heparin in 78 patients with large AMI undergoing primary PCI. Bivalirudin reduced the index of microcirculatory resistance but had no significant effect on infarct size, early CMR-assessed MVO or LVEF at 90 days compared with unfractionated heparin [325].

Intracoronary epinephrine can mediate coronary vasodilatation at lower doses and, consequently, has been used mostly to reverse CNR. Navarese et al. [326] tested intracoronary epinephrine in 30 consecutive patients with STEMI and established CNR (defined as TIMI flow grade 0–1 and myocardial blush grade 0–1). Intracoronary epinephrine restored a TIMI flow grade of 2 (64.3% vs. 12.5%) and 3 (28.6% vs. 18.8%) compared with patients who received conventional treatment. Intracoronary epinephrine significantly improved ST-segment resolution and LVEF and reduced the 30-day composite endpoint of death or heart failure. In the Comparison of Intracoronary Epinephrine and Adenosine for No-Reflow in Normotensive Patients With Acute Coronary Syndrome (COAR) trial that randomized 201 patients with ACS and CNR to intracoronary epinephrine or adenosine, intracoronary epinephrine improved final TIMI flow grade of three and final corrected TIMI frame count but not final myocardial blush grade or in-hospital or short-term mortality or MACE compared with adenosine [327]. Two observational studies showed that intracoronary epinephrine reversed CNR in 9 of 12 patients [328] and in 74 of 81 patients [329] with STEMI and persistent CNR after primary PCI. Although intracoronary epinephrine appears to reverse CNR, it may increase the risk of malignant arrhythmias and the quality of studies does not allow a firm recommendation for its use to treat CNR.

Suboptimal platelet response to dual antiplatelet therapy was associated with a greater extent of MVO [330]. This has raised interest in newer antiplatelet drugs, which cause a deeper and more predictable platelet inhibition. Subgroup analyses from the Study of Platelet Inhibition and Patient Outcomes (PLATO) [331] and Administration of Ticagrelor in the Cath Lab or in the Ambulance for New ST Elevation Myocardial Infarction to Open the Coronary Artery (ATLANTIC) [332] trials showed no benefit of ticagrelor vs. clopidogrel in improving reperfusion or reducing the frequency of CNR after primary PCI. The Reducing Micro Vascular Dysfunction in Acute Myocardial Infarction by Ticagrelor (REDUCE-MVI) trial that randomized 110 patients with STEMI to receive loading dose of ticagrelor or prasugrel showed no significant difference between the drugs in the index of microcirculatory resistance or its recovery over time. Intramyocardial hemorrhage was less frequent in patients who received ticagrelor but there was no significant difference in the infarct size on the one-month CMR imaging [333]. A 2018 meta-analysis of 14 randomized trials and one observational study with 4162 patients showed that ticagrelor significantly reduced the frequency of CNR after primary PCI and reduced the risk of MACE up to 180 days compared with clopidogrel with no significant increase in the risk for bleeding [334]. The efficacy of cangrelor to reduce MVO and infarct size in patients with STEMI is under investigation [335].

Over the years, a plethora of drugs and strategies, alone or in combination, have been used to protect microcirculation and prevent MVO or CNR and reduce infarct size in patients with STEMI. Cyclosporine A—an inhibitor of mitochondrial permeability transition pore opening—reduced infarct size in a small, randomized study of 58 patients with STEMI undergoing primary PCI [336]. However, two randomized trials showed no benefit of intravenous cyclosporine A in reducing infarct size (estimated by CMR or high-sensitivity cardiac troponin), ST-segment resolution, LVEF improvement or prevention of adverse remodeling of left ventricle or incidence of MACE at 6 months and 1 year [337,338]. Experimental studies suggested that hyperoxemia may reduce formation of lipid peroxide radicals, increase NO availability via expression of NO synthase and inhibit leukocyte adherence and plugging in microcirculation. The Acute Myocardial Infarction with Hyperoxemic Therapy (AMIHOT) trial showed that intracoronary supersaturated oxygen did not improve ST-segment resolution, regional wall motion by echocardiography or scintigraphic infarct size [339]. However, patients with STEMI of anterior wall undergoing reperfusion within the first 6 h showed improvement in regional wall motion and smaller infarct size. The AMIHOT-2 trial that included patients with STEMI of anterior wall showed that intracoronary delivery of supersaturated oxygen reduced infarct size with no difference in 30-day MACE compared with placebo [340]. However, a SWEDEHEART registry-based randomized trial showed no beneficial effects of routine supplemental oxygen in terms of one-year mortality in patients with suspected AMI and oxygen saturation ≥ 90% [341]. In the IntraCoronary Hyper-oxemic Supersaturated Oxygen Therapy (IC-HOT) study, delivery of supersaturated oxygen in the left main coronary artery for 60 min after PCI in patients with anterior STEMI was safe [342]. Although, hypothermia was hypothesized to reduce metabolic demand and inflammatory response, three randomized trials showed no beneficial effects of this therapeutic strategy in reducing the MVO or infarct size in patients with STEMI. Instead, an increase in the frequency of adverse events was observed [343,344,345]. However, the strategy of elective intracoronary hypothermia during the primary PCI remains under investigation and the preliminary results indicate that this strategy is safe [346]. Atrial natriuretic peptides suppress endothelin-1 production and activate reperfusion injury salvage kinase (RISK) cardioprotective pathway. Atrial natriuretic peptide agonist carperitide reduced enzymatic infarct size and improved LVEF in patients with STEMI undergoing primary PCI [347]. However, evidence on the beneficial effects of these drugs in reducing MVO and CNR remains limited. Preclinical research suggested that glucagon-like peptide-1 agonist exenatide may reduce infarct size and improve ventricular function by reducing apoptosis and oxidative stress. In one study with 172 patients with STEMI, intravenous exenatide increased myocardial salvage and reduced CMR-assessed infarct size compared with placebo (saline) [348]. In another study of 58 patients with STEMI, patients who received exenatide showed a significant reduction of the absolute mass of delayed hyperenhancement on CMR compared with control patients [349]. However, in a more recent and larger trial of 191 patients with STEMI, intravenous exenatide failed to reduce infarct size after correction for the area at risk in the acute phase or final infarct size as a percentage of the left ventricle at 4 months compared with placebo [350]. A 2009 meta-analysis of 17 studies by Iwakura et al. [351] showed that nicorandil—a hybrid of the K_ATP_ channel opener and nitrate—reduced the incidence of TIMI flow grade ≤ 2 by 37% and improved LVEF by 3.7% with no impact on peak creatine kinase. A multicenter, randomized, double-blind, dose-finding placebo-controlled study of ITF-1697—a C-reactive protein-derived tetrapeptide—showed no difference between 0.1 and 1.0 μg/kg/min study arms or placebo with respect to post-procedural TIMI flow, corrected TIMI frame count, blush grade and ST-segment resolution in 402 patients with AMI [352]. In a rat model, a combination DNase1 and recombinant tissue-type plasminogen activator reduced NET density, no-reflow area in the ischemic region and infarct size and ameliorated ischemia/reperfusion-induced adverse left ventricular remodeling [74]. The impact of current anti-inflammatory therapies used in patients with ACS on MVO and CNR remains largely untested [215].

Earlier studies with small numbers of patients suggested that ischemic postconditioning may reduce the infarct size estimated by CMR [353,354]. However, a 2014 meta-analysis of 15 randomized trials with 1545 patients showed no beneficial effects of ischemic postconditioning on ST-segment resolution, infarct size or any of the clinical outcomes (mortality, recurrent myocardial infarction, stent thrombosis or composite MACE) at 5 months after PCI [355]. The third Danish Study of Optimal Acute Treatment of Patients With ST Elevation Myocardial Infarction–Ischemic Postconditioning (DANAMI-3–iPOST) trial that randomized 1234 patients with STEMI presenting within 12 h from symptom onset and a baseline TIMI flow grade of 0–1 to conventional primary PCI or postconditioning (4 cycles of 30 s balloon occlusions followed by 30 s of reperfusion immediately after opening of the infarct-related artery and before stent implantation) found no difference in CMR-estimated infarct size, extent of MVO, myocardial salvage index or LVEF at 3 months or MACE (death or hospitalization for heart failure) over a median of 38 months [356]. Earlier studies also suggested that remote postconditioning applied during the primary PCI may improve myocardial salvage and ST-segment resolution, reduce infarct size, and lower MACE in patients with STEMI [357,358]. However, recent research did not support the benefits of remote postconditioning reported in earlier trials. The LIPSIA CONDITIONING trial that included 696 patients with STEMI showed that postconditioning alone did not improve myocardial salvage, infarct size or MVO [359] or MACE at 3.6 years [360]. However, combined ischemic postconditioning and remote postconditioning improved myocardial salvage [359] and MACE over a median of 3.6 years, mostly driven by reduced incidence of new congestive heart failure [360]. However, the CONDI-2/ERIC-PPCI trial that randomized 5401 patients with STEMI undergoing primary PCI to remote postconditioning or control, did not show a benefit of remote postconditioning in reducing MACE at one year (death or hospitalization for heart failure: 8.6% vs. 9.4%; *p* = 0.32) [361]. Representative therapeutic studies of CNR are shown in Table 2.

In summary, despite decades of intensive research and testing of numerous agents and therapeutic strategies targeting all known pathophysiological mechanisms of MVO and CNR, no satisfactory therapy has been found to prevent or reverse these phenomena and consistently improve the clinical outcome. Although there is no evidence to support the routine use of any above-mentioned therapeutic strategies during primary PCI, in patients with large thrombus burden at high risk for CNR, manual thrombus aspiration with direct stenting continues to be used to reduce distal embolization. Vasodilator therapy has been the mainstay of CNR therapy for almost 50 years in patients with AMI who develop persistent CNR and continues to be used in catheterization laboratories based on operator’s personal experience or intuition, albeit without evidence of a proven benefit. Even though vasodilator therapy may improve some markers of reperfusion (particularly angiographic markers), it has largely failed to reduce infarct size or improve clinical outcome. Powerful antithrombotic drugs like glycoprotein 2b/3a may be used but they increase the risk of intramyocardial hemorrhage.

## 7. Concluding Remarks

MVO and CNR are relatively frequent phenomena that develop following reperfusion therapy in patients with STEMI. The frequency of CNR or MVO after primary PCI differs widely depending on the sensitivity of diagnostic methods and timing of examination. Coronary angiography is readily available and most convenient to diagnose CNR but it is highly conservative due to suboptimal (too early) timing of examination (end of PCI procedure) and low sensitivity of angiographic criteria. CMR imaging is the most sensitive method to diagnose MVO and CMR that provides information on the presence, localization and extent of MVO. CMR imaging detects also intramyocardial hemorrhage and accurately estimates infarct size in clinical setting. MVO and CNR markedly negate the benefits of reperfusion therapy and contribute to poor clinical outcomes including adverse remodeling of left ventricle, worsening or new congestive heart failure and increased risk of mortality. Since MVO and CNR are important predictors of clinical outcome, patients with STEMI developing these phenomena are in need of careful risk stratification and tailored therapy. Despite almost 50 years of research and targeting of all known factors (and mechanisms) involved in the pathophysiology of MVO and CNR, no therapy has been found that prevents or reverses these phenomena (when they develop) and provides consistent clinical benefit in patients with STEMI. While no MVO or CNR preventive therapies can be recommended to be routinely used in patients with STEMI undergoing primary PCI, early reperfusion by primary PCI remains the only means to stop ongoing ischemia, reduce the infarct size and consequently reduce the occurrence and extent of MVO and CNR. For the time being, prevention or alleviation of MVO and CNR remain unmet goals in the therapy of STEMI that continue to be under intense research.

## Figures and Tables

**Table 1:** Frequency of coronary no-reflow by various diagnostic methods in patients with acute myocardial infarction.

Author (Year)	Patients	Reperfusion Strategy	Diagnostic Method/CNR Criteria	Frequency of CNR or MVO
Rezkalla et al. [170] (2010)	347 patients with STEMI	Primary PCI	Coronary angiography TIMI < 3; MBG < 3	32% (TIMI) 57% (MBG)
Hamada et al. [176] (2001)	104 patients with first AMI	Primary PCI with TIMI flow 3 restoration	Coronary angiography Slow (≤23) CTFC	43%
van ‘t Hof et al. [178] (1998)	777 patients with STEMI	Primary PCI	Coronary angiography MBG 0–1	30%
Henriques et al. [171] (2003)	924 patients with STEMI	Primary PCI with TIMI flow 3 restoration	Coronary angiography MBG 0–1	11%
Appelbaum et al. [188] (2009)	31 patients with STEMI	Primary PCI	Coronary angiography TMPG < 3	52%
Ndrepepa et al. [185] (2010)	1406 patients with STEMI	Primary PCI	Coronary angiography TIMI < 3; TIMI 3 with TMPG 0–1	29%
Ito et al. [193] (1996)	126 patients with first STEMI of anterior wall	Primary PCI or thrombolysis	MCE Residual contrast defects within area at risk	37%
Galiuto et al. [133] (2003)	24 patients with first STEMI	Primary PCI or thrombolysis	MCE Reduced or absent opacification	66%
Taylor et al. [201] (2004)	20 patients with STEMI	Primary PCI	CMR Delayed wash-in of gadolinium	95%
De Waha et al. [202] (2017)	1688 patients with STEMI	Primary PCI	CMR Late gadolinium enhancement	57%

AMI = acute myocardial infarction; CMR = cardiac magnetic resonance; CNR = coronary no-reflow; CTFC = corrected TIMI frame count; MBG = myocardial blush grade; MCE = myocardial contrast echocardiography; MVO = microvascular obstruction; PCI = percutaneous coronary intervention; TIMI = Thrombolysis in myocardial infarction; TMPG = TIMI myocardial perfusion grade.

**Table 2:** Therapeutic strategies used to prevent or treat coronary no-reflow.

Author (Year)	Type of Study	Patients	Reperfusion Strategy	Coronary No-Reflow/Clinical Outcome
Stone et al. [109] (2005)	RT (PCI + distal protection or PCI alone)	501 patients with STEMI	Primary PCI	Complete STR: 63% vs. 62% 6-month MACE: 10% vs.11%
Jolly et al. [288] (2015)	RT (PCI + aspiration thrombectomy or PCI alone)	10,732 patients with STEMI	Primary PCI	CNR: 2.4% vs. 2.8% (*p* = 0.28) 6-month adverse events: 6.9% vs. 7.0%
Lønborg et al. [290] (2017)	RT (deferred or immediate stenting)	510 patients with STEMI	Primary PCI	MVO: 43% vs. 42% (*p* = 0.78) Myocardial SI: 66% vs. 67% (*p* = 0.80)
Stone et al. [292] (2012)	RT (mesh-covered stent or conventional stents)	433 patients with STEMI	Primary PCI	TIMI 3: 91.7% vs. 82.9% (*p* = 0.006) STR ≥ 70%: 57.8% vs. 44.7% (*p* = 0.008) MBG: 83.9% vs. 84.7% (*p* = 0.81)
van‘t Hof et al. [296] (2008)	RT (prehospital high-bolus dose tirofiban or placebo)	936 patients with STEMI	Primary PCI	Complete STR (>70%): 65.6% vs. 60.0% (*p* = 0.08)
Eitel et al. [172] (2013)	RT (intracoronary or intravenous abciximab)	2065 patients with STEMI	Primary PCI	Myocardial SI: 52% vs. 50% (*p* = 0.25) Late MVO: 49% vs. 47% (*p* = 0.19) IMH: 35% vs. 32% (*p* = 0.19)
Niccoli et al. [305] (2013)	RT (intracoronary adenosine or nitroprusside or placebo)	240 patients with STEMI and TIMI grade 0–1	Primary PCI	STR >70: 71% vs. 51% (adenosine vs. placebo; *p* = 0.009) Angiographic MVO: 18% vs. 30% (adenosine vs. placebo; *p* = 0.06)
Nazir et al. [307] (2016)	RT (intravenous adenosine or sodium nitroprusside or standard PCI)	247 patients with STEMI	Primary PCI	STR >70%: 68.3% vs. 65.1% (*p* = 0.66) Late MVO: 68.3% vs. 56.9% (*p* = 0.205) for adenosine vs. standard PCI
Siddiqi et al. [309] (2014)	RT (sodium nitrite infusion or placebo)	229 patients with STEMI	Primary PCI	Median infarct size at 6–8 days: 22% (nitrite) vs. 20% (placebo); (*p* = 0.30)
Roolvink et al. [319] (2016)	RT (intravenous metoprolol or placebo)	683 patients with STEMI	Primary PCI	Infarct size by CMR: 15.3% vs. 14.9% (*p* = 0.616)
Kim et al. [320] (2010)	RT (80-mg or. 10-mg atorvastatin)	171 patients with STEMI	Primary PCI	CTFC (80 mg vs.10 mg): 26.9 vs.34.1 (*p*= 0.01) MBG: 2.2 vs. 1.9 (*p* = 0.02) STR >70%: 39.5% vs. 23.8% (*p* = 0.03)
McCartney et al. [323] (2019)	RT (intracoronary alteplase 20 mg, alteplase 10 mg, or placebo)	440 patients with STEMI	Primary PCI	MVO by CMR (3.5% (20 mg) vs. 2.6% (10 mg) vs. 2.3% (placebo): All *p* values =NS
Navarese et al. [326] (2021)	Observational study (intracoronary epinephrine or no epinephrine)	30 patients with persistent CNR	Primary PCI	TIMI grade 3, 2 and 0–1: 28.6%, 64.3% 7.1%(epinephrine) and vs. 18.8%, 12.5% and 68.8% (no epinephrine); *p* = 0.004
Stone et al. [340] (2009)	RT (intracoronary supersaturated oxygen or control)	301 patients with STEMI of anterior wall	Primary PCI	Scintigraphic infarct size: 26.5% vs. 20% (adjusted *p* = 0.03) 30-day MACE: 5.4% vs. 3.8% (*p* = 0.77)
Roos et al. [350] (2016)	RT (intravenous exenatide or placebo)	191 patients with STEMI	Primary PCI	Infarct size by CMR: 18.8% vs. 18.8% (*p* = 0.96)
Hausenloy et al. [361] (2019)	RT (remote ischemic conditioning or standard treatment)	5401 patients with STEMI	Primary PCI	Death of hospitalization for heart failure: 8.6% vs. 9.4% (*p* = 0.32)

CMR = cardiac magnetic resonance; CNR = coronary no-reflow; CTFC = corrected TIMI frame count; IMH = intramyocardial hemorrhage; MACE = major adverse cardiovascular events; MBG = myocardial blush grade; MVO = microvascular obstruction; NS = nonsignificant; PCI = percutaneous coronary intervention; RT = randomized trial; SI = salvage index; STEMI = ST-segment elevation myocardial infarction; STR = ST-segment resolution; TIMI = thrombolysis in myocardial infarction.

## Data Availability

Not applicable.
